# Association of Visit-to-Visit Variability in Fasting Plasma Glucose with Digestive Cancer Risk

**DOI:** 10.1155/2022/4530894

**Published:** 2022-07-13

**Authors:** Nan Zhang, Yueying Wang, Gary Tse, Guangping Li, Shouling Wu, Tong Liu

**Affiliations:** ^1^Tianjin Key Laboratory of Ionic-Molecular Function of Cardiovascular Disease, Department of Cardiology, Tianjin Institute of Cardiology, Second Hospital of Tianjin Medical University, Tianjin 300211, China; ^2^Epidemiology Research Unit, Cardiovascular Analytics Group, Hong Kong, China-UK Collaboration, China; ^3^Kent and Medway Medical School, Canterbury, Kent CT2 7NZ, UK; ^4^Department of Cardiology, Kailuan Hospital, North China University of Science and Technology, Tangshan City, China

## Abstract

**Background and Aims:**

The aim of this study is to investigate the association between visit-to-visit variability in fasting plasma glucose (FPG) and the risk of digestive cancers among individuals with and without diabetes.

**Methods:**

Using data from Kailuan cohort, a prospective population-based study, individuals who had at least two measurements of FPG between 2006 and 2012 without prior cancer were included in this study. Four indexes of variability were used, including standard deviation (SD), coefficient of variation (CV), variability independent of the mean (VIM), and average successive variability (ARV). Cox regression was used to evaluate the relationship between the quartiles of FPG variability and digestive cancers.

**Results:**

A total of 98,161 individuals were studied. Over a mean follow-up of 9.32 ± 0.81 years, 1103 individuals developed incident digestive cancer (1.21 per 1000 person-years). Compared to the individuals in the lowest quartile, those in the highest quartile of FPG variability by SD had 38.7% higher risk of developing overall digestive cancers after adjusting for the significant confounders (hazard ratio, 1.387; 95% confidence interval, 1.160-1.659; *P* = 0.0003). Higher FPG variability was associated with significantly higher risks of colorectal cancer (fully adjusted HR 1.432, 95% CI [1.073-1.912], *P* = 0.015) and pancreatic cancer (fully adjusted HR 2.105, 95% CI [1.024-4.329], *P* = 0.043), but not liver cancer (fully adjusted HR 1.427, 95% CI [0.973-2.092], *P* = 0.069) or esophageal and gastric cancer (fully adjusted HR 1.139, 95% CI [0.776-1.670], *P* = 0.506). Subgroup analyses showed that individuals who were younger (<65 years), male, and those without diabetes experienced a predominantly higher risk of developing digestive cancers. Similar results were observed when using CV, VIM, and ARV.

**Conclusions:**

FPG variability was significantly associated with increasing risk of digestive cancers, especially for pancreatic and colorectal cancer. Our study suggested a potential role of FPG variability in risk stratification of digestive cancers. Approaches that reduce FPG variability may lower the risks of incident digestive cancers among the general population. This trial is registered with ChiCTR-TNRC-11001489.

## 1. Introduction

Cancer is a leading cause of death, imposing a substantial burden on public health systems and social economies globally. According to the global cancer statistics 2020 [1], there are 19.3 million new cancer cases and almost 10.0 million cancer deaths in 2020 worldwide, among which the digestive cancers account for a large proportion. The morbidity and mortality of colorectal, stomach, liver, and esophageal cancer rank among the top ten cancers, and pancreatic cancer ranks as the seventh leading cause of cancer-related death worldwide [1]. The situation in China has also been alarming. According to the recently released data, China has lower cancer incidence but a 30% and 40% higher cancer mortality than the UK and USA, among which 36.4% of the cancer-related deaths were from digestive cancers [2]. The high burden of digestive cancers highlights the need for identification of efficient modifiable risk factors to foster the prevention of these multifactorial pathologies [3].

In addition to established risk factors, such as smoking, alcohol abuse, obesity, physical inactivity, and unhealthy diet [4], there is increasing body of evidence showing that type 2 diabetes mellitus (T2DM) increases the risks of several digestive cancers, including colorectal, gallbladder, liver, and pancreatic cancer [5]. Fasting plasma glucose (FPG) has been associated with digestive cancer incidence in a dose-response manner across the range of prediabetic and diabetic states [6–8]. Oscillating levels of glucose may have a more damaging effect than a constantly high level of glucose, which could have a deleterious effect not merely on the onset and progression of diabetes complications [9] but also in clinical conditions other than diabetes [10]. Glycemic variability has been used to determine the fluctuations of blood glucose over continuous or intermittent time intervals [11] and has become an important marker of glycemic control. The detrimental effects of glycemic variability on target organs can be realized through oxidative stress, endothelial dysfunction, and chronic inflammation [10, 12]. Interestingly, these mechanisms are also implicated in the pathophysiology of cancer, implying that higher glycemic variability might increase the risk of cancer [13]. While previous retrospective studies have demonstrated that long-term FPG variability was a strong predictor of overall cancer incidence in T2DM patients [14] and overall cancer mortality in patients with and without T2DM, [14, 15] however, to the best of our knowledge, no published study has addressed the relationship between glycemic variability and digestive cancer incidence in individuals with and without T2DM up to date.

In this prospective cohort study, we aim to evaluate the associations of visit-to-visit glycemic variability with the risk of overall and individual types of digestive cancers among participants with and without T2DM.

## 2. Methods

### 2.1. Population

Participants were from the Kailuan study, and details about study procedures have been detailed elsewhere [16]. Briefly, Kailuan study is a prospective, population-based cohort, which was conducted at the Kailuan General Hospital and ten affiliated hospitals in the city of Tangshan, Hebei Province, China. Since 2006, participants from the Kailuan community have completed questionnaire interview and the first survey in the 11 hospitals. Following surveys were conducted every 2 years.

In this study, the target population included all adult who (1) had at least two health examinations biennially and (2) without a prior cancer diagnosis or missing FPG data. The study was performed according to the guidelines of the Declaration of Helsinki and was approved by the Ethics Committee of the Kailuan General Hospital (Approval Number: 2006-05). All participants provided written informed consent.

### 2.2. Data Collection

Face-to-face interview was conducted by trained nurses or physicians using standard questionnaires to collect demographic information such as age, sex, smoking status, alcohol use, comorbidities, and medical history. Body weight and height were measured by trained nurses while participants were wearing light clothes without shoes, and the body mass index (BMI) was calculated according to the formula that BMI = weight (kg)/height (m^2^). The overnight fast blood samples of the participants were obtained for the measurement of FPG. All blood measurements were conducted using an automatic analyzer in the central laboratory of the Kailuan General Hospital by standardized operating procedure and were subjected to regular quality control.

### 2.3. Assessment of Fasting Plasma Glucose Variability

FPG variability was defined as intraindividual visit-to-visit variability during at least a 4-year period prior to the baseline. Considering that there are no gold standard measures of FPG variability, we applied four variability indexes that have been described previously [15, 17], to capture different aspects of FPG variability:

1. Standard deviation $\left(\text{SD}\right)=\sqrt{\left(1/\left(n-1\right)\right)\sum_{i=1}^{n}{\left(x_{i}-\bar{x}\right)}^{2}}$
2. Coefficient of variation (CV) = (SD/*x* × 100%)
3. Variability independent of the mean (VIM) = 100 × SD/mean^*β*^ (*β*: the regression coefficient based on natural logarithm of SD on natural logarithm of the mean)
4. Average real variability (ARV) = (1/(*N* − 1))∑_*K*=1_^*N*−1^|Value_*K*+1_ − Value_*K*_|∑

### 2.4. Assessment of Digestive Cancers

The participants were followed from the baseline until the diagnosis of digestive cancer, death, or until December 31, 2019. The method for diagnosis of digestive cancers in Kailuan study was described previously [18]. Incident digestive cancer cases were obtained by self-reporting every two years and from medical linkage with the Tangshan medical insurance system, the Kailuan social security system, or discharge lists from the 11 affiliated hospitals. All cases were confirmed through medical records reviewed by clinical experts [16, 18]. Digestive cancers (C15-C26) were coded according to the *International Classification of Diseases* (ICD), Tenth Revision. The site-specific cancers included biliary (C23 and C24), liver (C22.0 and C22.2-C22.9), pancreatic (C25), esophageal (C15), gastric (C16), and colorectal (C18-C21) cancers. Death certificates were also obtained from the Kailuan social security system annually [16, 18]. The Tangshan medical insurance system and the Kailuan social security system cover all the participants' health information and living status.

### 2.5. Statistical Analysis

Categorical data are presented as frequencies and percentages and compared between groups using chi-squared tests. Continuous data are reported as mean and SD or, in the case of skewed distribution, as median and IQR and compared using one-way analysis of variance (ANOVA). Visit-to-visit FPG variability was assessed as both quartiles and a continuous parameter. Person-years were calculated from the date of the first interview to the date of digestive cancer diagnosis, death, or the date of last interview, whichever came first. Multiple imputation method was used to handle the missing data of the baseline covariates.

Multivariable Cox proportional hazards regression models were applied to calculate HRs and 95% confidence intervals (CI) for the association between FPG variability and incidence digestive cancers. Several models were constructed using different measures of FPG variability (SD, CV, VIM, and ARV) with sequential adjustment for potential confounders as follows: model 1: age and gender; model 2: model 1 plus diabetes mellitus, hypertension, antihypertensive drugs, hypoglycemic drugs, baseline FPG, and LDL-C; and model 3: model 2 plus BMI, smoking, alcohol status, physical activity, and family history of cancer. Subgroup analyses were conducted according to age (<65 years and ≥65 years), sex, history of DM, and BMI (<25 kg/m^2^ and ≥25 kg/m^2^). All analyses were performed using SAS statistical software, version 9.4 (SAS Institute). *P* < 0.05 was considered statistically significant.

## 3. Results

### 3.1. Baseline Characteristics

**Figure 1:**
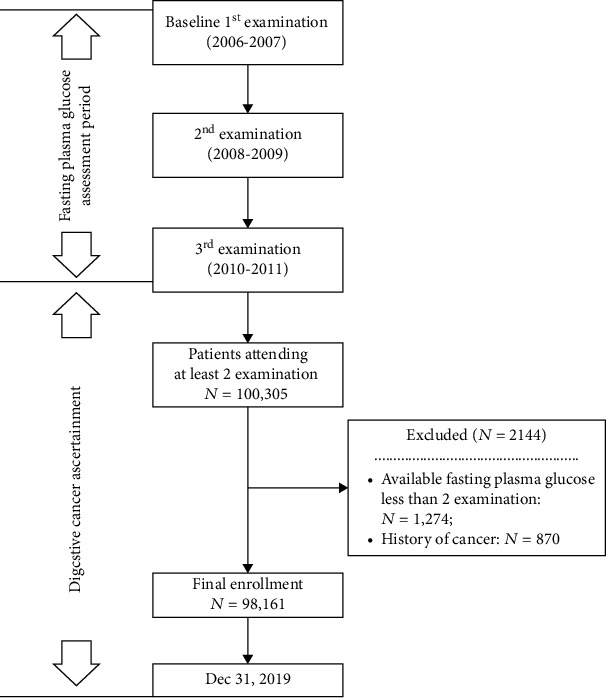
Selection of the study population.

Individuals who had at least two health examinations biennially were eligible for the evaluation of FPG variability (*n* = 100,305). Participants were excluded if they had a prior cancer diagnosis (*n* = 870) or missing FPG data (*n* = 1274). Finally, a total of 98,161 participants (mean age, 53.62 ± 12.35 years; 78.94% male) were included in this study (Figure 1). The median (IQR) variability of FPG, as measured by SD, was 0.43 (0.24, 0.71) mmol/L. Over a mean follow-up duration of 9.32 ± 0.81 years, 1103 participants developed incident digestive cancer (1.21 per 1000 person-years). Among them, 419 (37.99%) patients were diagnosed with colorectal cancer, 243 (22.03%) with liver cancer, 225 (20.40%) with gastric cancer, 116 (10.52%) with esophageal cancer, 83 (7.52%) with pancreatic cancer, and 17 (1.54%) with biliary cancer (Figure S1).

The baseline characteristics of participants stratified by quartiles of visit-to-visit FPG variability assessed by SD are presented in Table 1, and those according to CV, VIM, and ARV are presented in Tables S1–S3. Participants with higher quartiles of FPG variability (quartile 4) were more likely to be older, male, more likely to have comorbidities such as hypertension, T2DM, dyslipidemia, and CKD, and more likely to have family history of cancer. These participants also had higher rate of consuming antihypertensive, hypoglycemic, and lipid-lowering agents and higher level of BMI, blood pressure, FPG, total cholesterol, and triglyceride. As for lifestyle characteristics, subjects in the 3^rd^ quartile of FPG variability presented with higher rate of currently smoking and drinking and lower rate of regular exercising and lower income.

### 3.2. FPG Variability and the Risk of Total Digestive Cancers

The incidence rate of overall digestive cancer increased in a graded fashion across quartiles of SD (0.92 per 1000 person-years in quartile 1 and 1.51 per 1000 person-years in quartile 4). The associations between FPG variability and digestive cancer assessed by SD are showed in Table 2. Compared with the lowest quartile of FPG variability, patients in the highest quartile presented with significantly higher risk of developing digestive cancer after adjusting for age and sex (model 1, HR 1.425, 95% CI [1.201-1.692]; *P* < 0.0001), which was consistent after additionally adjusting for LDL-C, baseline FPG, hypertension, DM, antihypertensive drugs, and hypoglycemic drugs (model 2, HR 1.391, 95% CI [1.163-1.663]; *P* = 0.0003). The association remained significant in the fully adjusted model (model 3) which further accounted for the BMI, smoking status, drinking status, physical exercise, and family history of cancer (HR 1.387, 95% CI [1.160-1.659]; *P* = 0.0003). The association between FPG variability and digestive cancer was also significant in quartiles 2 and 3; patients in quartile 3 seemed to have higher risk of incident digestive cancer (with the highest HR). An increase in the quartile of SD was significantly associated with increased risk of digestive cancer across three models (all *P* for trend <0.0001). Similar patterns of association were also observed for other measures of FPG variability (CV, VIM, and ARV), as presented in Tables S4–S6.

### 3.3. FPG Variability and Site-Specific Digestive Cancer

Compared with the lowest quartile, the risk of new-onset colorectal cancer was significantly higher in quartile 3 and quartile 4 of FPG variability assessed by SD, VIM, and CV and in quartile 3 of ARV, which was consistent across three models. In the fully adjusted model (model 3), the association between incident colorectal cancer and FPG variability was significant as assessed by SD (Q3, HR 1.600, 95% CI [1.207-2.121], *P* = 0.001; Q4, HR 1.432, 95% CI [1.073-1.912], *P* = 0.015), VIM (Q3, HR 1.607, 95% CI [1.221-2.115], *P* = 0.0007; Q4, HR 1.400, 95% CI [1.039-1.887], *P* = 0.027), CV (Q3, HR 1.426, 95% CI [1.073-1.896], *P* = 0.015; Q4, HR 1.400, 95% CI [1.059-1.851], *P* = 0.018) and ARV (Q3, HR 1.390, 95% CI [1.056-1.830], *P* = 0.019).

Individuals in the highest quartile of FPG-SD had approximately 2-fold higher risk of developing pancreatic cancer compared with the lowest quartile, across three models (model 1, HR 2.207, 95% CI [1.101-4.425], *P* = 0.026; model 2, HR 2.090, 95% CI [1.017-4.294], *P* = 0.045; model 3, HR 2.105, 95% CI [1.024-4.329], *P* = 0.043).

Compared with the lowest quartile, the association between liver cancer and SD of FPG in quartile 4 was significantly higher in model 1 (HR 1.470, 95% CI [1.016-2.127], *P* = 0.040), whereas this association did not reach statistical significance in model 2 (HR 1.441, 95% CI [0.984-2.111], *P* = 0.061) and model 3 (HR 1.427, 95% CI [0.973-2.092], *P* = 0.069). In addition, subjects in quartile 3 of FPG variability also presented with significantly higher risk of incident liver cancer, across three models. As for gastric and esophageal cancer, the association between incident risk and FPG variability among individuals with higher quartiles did not reach the traditional statistical significance (model 3, HR 1.139 for Q4 vs. Q1, 95% CI [0.776-1.670], *P* = 0.506). The absence of significantly increased risk of gastric and esophageal cancer was consistent across three models and four measures of FPG variability. The associations between FPG variability and site-specific digestive system cancer assessed by SD are showed in Table 3, and those according to CV, VIM, and ARV are presented in Tables S7–S9.

### 3.4. Subgroup Analyses

**Figure 2:**
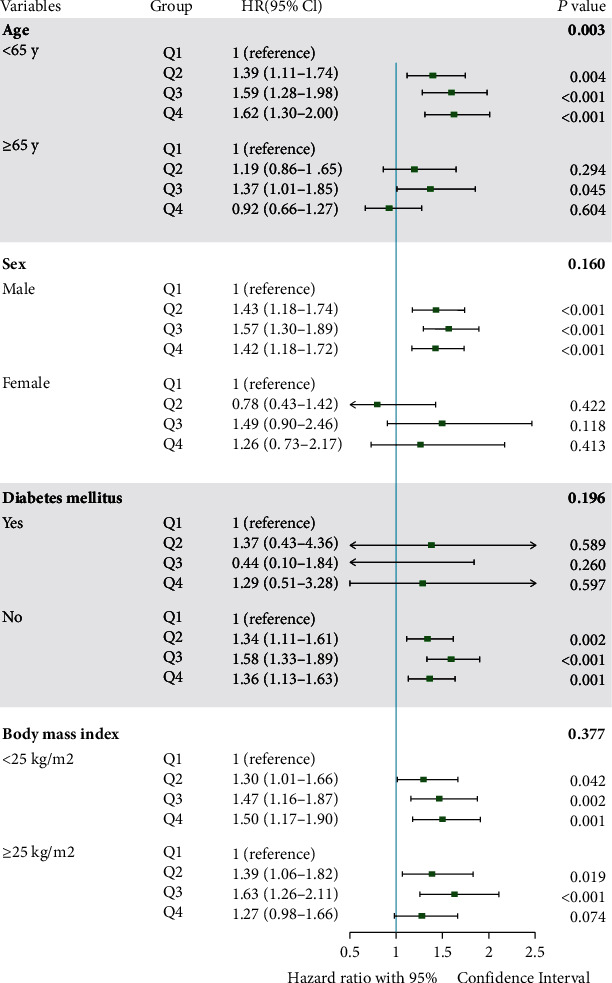
Hazard ratio for incident digestive cancers in different subgroups among the overall population by SD quartile. Subgroup analyses by age, gender, diabetes mellitus, and body mass index were presented. In this analysis, HR was adjusted for age, gender, low-density lipoprotein cholesterol, baseline fasting plasma glucose levels, diabetes mellitus, hypertension, body mass index, antihypertensive medications, antidiabetic medications, current smoking, drinking, physical exercise, and family history of cancer (model 3). Bold values were the *P* values for interaction. Abbreviations: CI: confidence interval; HR: hazard ratio.

In subgroup analysis according to age, the association between FPG variability assessed by SD and digestive cancers was more significant among individuals < 65 years, compared with those ≥ 65 years (*P*_interaction_ = 0.003). Additionally, male subgroup rather than female presented with significant association between FPG variability and risk of digestive cancers, across three models. In subgroup analysis according to DM, those without DM confronted with significantly higher risk of developing digestive cancers. In subgroup analysis according to BMI, the association between FPG variability and the risk of digestive cancers was significant across two BMI subgroups (Table S10). There was no interaction by gender, DM, or BMI on the association of FPG variability with incident digestive cancers (*P*_interaction_ < 0.05) (Figure 2). The results of subgroup analyses between FPG variability and digestive cancers according to CV, VIM, and ARV are showed in Tables S11–S13, which are consistent with those according to SD.

## 4. Discussion

In this large, prospective, population-based cohort study, several key findings were noted. First, FPG variability was significantly associated with risk of digestive cancers among patients with and without T2DM, independent of potential confounders. Second, higher FPG variability may increase the risk of incident colorectal and pancreatic cancer, whereas a significant association was not observed for liver cancer or gastric and esophageal cancer. Third, the association between FPG variability and digestive cancer was more significant among individuals who were <65 years, male, or without baseline T2DM.

Epidemiologic evidence suggests that individuals with diabetes are at a significantly higher risk of many forms of cancer. According to the 2010 American Diabetes Association and the American Cancer Society Consensus Report, the relative risks imparted by diabetes are approximately 2-fold or higher for cancers of the liver and pancreas and approximately 1.2-fold to 1.5-fold for cancers of the colon/rectum, whereas the potential biologic links between diabetes and cancers are incompletely understood [19]. Persistent hyperglycemia is a predominant characteristic of diabetes, which also has been proposed as one of potential biologic links between diabetes and cancer risks. Accumulating studies have reported a significant association between higher blood glucose concentration and risk of digestive cancers, such as colorectal, pancreatic, liver, and gastric cancer [6–8, 20]. Glycemic variability is an integral component of glucose homoeostasis, which can represent the presence of excess glycemic excursions (hyperglycemic spikes and hypoglycemic episodes) [21]. High glycemic variability seems to exert more detrimental effects than persistent hyperglycemia on the pathogenesis of diabetic cardiovascular complications [10]. Previously, glycemic variability has been used to predict the diabetic complications, cardiovascular adverse events, and mortality among individuals with or without diabetes [21–24]. However, whether glycemic variability could serve as an independent risk factor for digestive cancers remained unknown.

Several existing studies have preliminarily explored the potential prediction value of glycemic variability in the cancer settings. A recent retrospective cohort study which enrolled 30,026 individuals demonstrated that long-term fluctuation of FPG assessed by CV was significantly associated with cancer mortality and all-cause mortality in the general population [15]. In the Taichung Diabetes Study which retrospectively analyzed 4805 patients, annual variability of FPG was also significantly predictive of overall cancer incidence and mortality in patients with T2DM [14]. However, to the best of our knowledge, only two Korean studies have specifically focused on the association between glycemic variability and digestive cancer. Jun et al. using the Korean NHIS-Health Screening Cohort which included 246,241 nondiabetic individuals identified a significant association between FPG variability and increased risk of colorectal cancer [25]. Hong et al. have focused on the gastric cancer incidence and demonstrated that high variability in visit-to-visit FPG levels was independently associated with an increased risk of gastric cancer in a DM-free population [26]. Compared with these two studies which only conducted in nondiabetic populations and focused on specific digestive cancer, our study has explored the association between FPG variability and several major types of digestive cancers among individuals with and without diabetes. In line with the previous study [25], our results also suggested a significant association between FPG variability and colorectal cancer among diabetic and nondiabetic population. Inconsistently with Hong et al. that conducted in nondiabetic population [26], our study failed to demonstrate a significant association between FPG variability and gastric and esophageal cancer among individuals with and without diabetes, which may be, at least partially, attributed to the relatively low event number, regional and population disparity. In addition, our study further adds evidence to the significant association between FPG variability with overall digestive cancers and pancreatic cancer in individuals with and without diabetes. Further investigation into the potential effects of FPG variability on different types of digestive cancer and the underlying mechanisms is of utmost importance.

The mechanisms underlying the observed associations between FPG variability and digestive cancers remain incompletely understood. Several possible mechanisms may explain our findings. Firstly, increased glycemic variability can be a powerful inducer of oxidative stress in both healthy subjects and diabetic patients [27]. The excessive reactive oxygen species can activate prooncogenic signaling pathways such as receptor tyrosine kinase (RTK), phosphatidylinositol 3-kinase (PI3K)/AKT, and nuclear factor kappa-light-chain-enhancer of activated B cells (NF-*κ*B), thereby aiding in cancer growth during early stages of tumorigeneses [28]. Secondly, oscillations in glucose levels could contribute to chronic inflammation, resulting in a rise of the levels of inflammatory cytokines such as IL-6 and tumor necrosis factor *α* [27]. Inflammation may predispose to the development of cancer and promote all stages of tumorigenesis [29]. Thirdly, oxidative stress and inflammation subsequently may lead to endothelial dysfunction, which in turn could mediate the development of cancer [13]. Fourthly, fluctuating glucose levels have been associated with irregular eating habits, inappropriate dietary patterns and nutritional factors, such as inappropriate carbohydrate quantity and quality, low protein and fiber intake [30]. Diet and nutrition can influence the risk of developing cancer, especially digestive cancers [31]. Other possible hypotheses such as mitochondria dysfunction and epigenetic modifications might also contribute to the significant association between glycemic variability and digestive cancers [27, 32, 33].

In our study, younger individuals (<65 years) presented with a more significant association between FPG variability and digestive cancers than older ones (≥65 years), which may be due to the fact that the multimorbidity and polypharmacy of the elderly might weaken the effect of FPG variability; another possible explanation is that younger individuals may suffer from longer duration of glucose fluctuation. Additionally, a significant association between FPG variability and increased risk of digestive cancers was observed only in men but not in women, which was consistent with the results of Jun et al. that higher FPG variability correlated with the risk of colorectal cancer only in the male subgroup [25]. Differing exposures to sex hormones may contribute to this sex disparity; [34, 35] besides, this observation may also suggest that men are more susceptible to digestive cancers than women if they have higher FPG variability [25]. When analyzed according to baseline DM, our study demonstrated a more predominant effect of FPG variability on digestive cancers among nondiabetic participants than those with diabetes, which may attribute to a number of factors, including that the effects of glycemic variability on the risk of cancer might be blunted by chronic hyperglycemia in subjects with diabetes, as well as some hypoglycemic agents such as metformin [36], thiazolidinediones [37], and sodium-glucose cotransporter-2 inhibitor [38] may exert beneficial effects on preventing digestive cancers. These factors may also help to explain that the highest HR was not observed in quartile 4 but in quartile 3, since the vast majority of diabetic patients were in quartile 4. Another possible reason is that subjects in quartile 3 presented with higher rate of smoking and drinking and lower rate of regular exercising, which may promote the development of cancer. Even if we have adjusted these potential confounders, given the nature of an observational study, we could not completely rule out the influence of these factors. A nongraded increment of HR of colorectal cancer among quartiles of FPG variability was also observed in Jun et al. [25]

## 5. Limitations

There are several limitations in our study. First, given the nature of the observational study, our study could not test the causal relationship between FPG variability and digestive cancers but only test associations. Second, although we have adjusted for a large number of potential confounding variables, we cannot completely rule out the possibility of other unknown confounders. Third, given the small event number, the association between FPG variability and some uncommon digestive cancers, such as cancers of the mouth and small intestine, was not analyzed in this study.

## 6. Conclusions

FPG variability was significantly associated with increasing risk of digestive cancers, especially for colorectal and pancreatic cancer and among those who were younger, male, and without diabetes. Our study provided evidence for a potential role of FPG variability in risk stratification of digestive cancers and also implicated that reducing FPG variability might help prevent the development of digestive cancers among individuals with and without diabetes. Future studies are warranted to further investigate the underlying mechanisms of the deleterious effects of FPG variability on digestive cancers.

## Figures and Tables

**Table 1:** Baseline characteristics according to quartiles of FPG variability assessed by SD.

Variables	Total *N* = 98,161	Quartile 1 *N* = 24,713	Quartile 2 *N* = 24,651	Quartile 3 *N* = 24,243	Quartile 4 *N* = 24,554	*P* value
Age (y)	53.62 ± 12.35	51.97 ± 12.46	52.50 ± 12.41	53.83 ± 12.40	56.19 ± 11.67	<0.01
Male sex, *n* (%)	77,492 (78.94)	19,078 (77.20)	18,969 (76.95)	19,319 (79.69)	20,126 (81.97)	<0.01
Comorbidities						
Hypertension, *n* (%)	15,471 (15.76)	3203 (12.96)	3429 (13.91)	3807 (15.70)	5032 (20.49)	<0.01
Diabetes mellitus, *n* (%)	4383 (4.47)	325 (1.32)	325 (1.32)	533 (2.20)	3200 (13.03)	<0.01
Dyslipidemia, *n* (%)	4638 (4.72)	933 (3.78)	1031 (4.18)	1179 (4.86)	1495 (6.09)	<0.01
^∗^CKD, eGFR < 60 mL/min, *n* (%)	8354 (8.51)	1975 (7.99)	1891 (7.67)	1973 (8.14)	2515 (10.24)	<0.01
Family history of cancer	3919 (3.99)	898 (3.63)	980 (3.98)	981 (4.05)	1060 (4.32)	<0.01
Medications						
Antihypertensive agents, *n* (%)	14,387 (14.66)	2917 (11.80)	3171 (12.86)	3546 (14.63)	4753 (19.36)	<0.01
Hypoglycemic agents, *n* (%)	3799 (3.87)	271 (1.10)	265 (1.08)	458 (1.89)	2805 (11.42)	<0.01
Lipid-lowering agents, *n* (%)	978 (1.00)	155 (0.63)	203 (0.82)	237 (0.98)	383 (1.56)	<0.01
Lifestyle						
Current smoker, *n* (%)	33,502 (34.13)	8290 (33.55)	8131 (32.98)	8521 (35.15)	8560 (34.86)	<0.01
Current alcohol use, *n* (%)	34,711 (35.36)	8822 (35.70)	8557 (34.71)	8815 (36.36)	8517 (34.69)	<0.01
Regular exercise, *n* (%)	68,808 (70.10)	17,611 (71.26)	17,270 (70.06)	16,848 (69.50)	17,079 (69.56)	<0.01
Low-income level, *n* (%)	51,315 (52.28)	13,401 (54.23)	12,717 (51.59)	12,388 (51.10)	12,809 (52.17)	<0.01
Health examination						
Body weight (kg)	71.16 ± 11.29	70.54 ± 11.29	70.81 ± 11.34	71.09 ± 11.18	72.21 ± 11.27	<0.01
BMI (kg/m^2^)	25.09 ± 3.40	24.84 ± 3.36	24.96 ± 3.35	25.05 ± 3.37	25.50 ± 3.47	<0.01
RHR (bpm)	73.63 ± 10.50	72.97 ± 10.01	72.91 ± 10.07	73.43 ± 10.39	75.24 ± 11.33	<0.01
SBP (mmHg)	132.03 ± 20.14	129.78 ± 20.03	130.14 ± 19.58	132.25 ± 19.91	135.97 ± 20.42	<0.01
DBP (mmHg)	84.72 ± 11.17	83.94 ± 11.15	84.01 ± 11.06	84.88 ± 11.16	86.06 ± 11.16	<0.01
FPG (mmol/L)	5.69 ± 1.85	5.22 ± 0.73	5.27 ± 0.77	5.45 ± 0.93	6.83 ± 3.16	<0.01
TC (mmol/L)	5.01 ± 1.38	4.93 ± 1.12	4.96 ± 1.37	5.02 ± 1.58	5.12 ± 1.39	<0.01
^†^TG (mmol/L)	1.29 (0.92, 1.92)	1.23 (0.87, 1.81)	1.24 (0.89, 1.83)	1.30 (0.92, 1.89)	1.40 (1.01, 2.13)	<0.01
LDL-C (mmol/L)	2.62 ± 1.07	2.61 ± 0.91	2.62 ± 1.07	2.62 ± 1.20	2.63 ± 1.11	0.06
HDL-C (mmol/L)	1.54 ± 0.53	1.53 ± 0.52	1.54 ± 0.50	1.55 ± 0.51	1.54 ± 0.58	<0.01
Scr (*μ*mol/L)	82.64 ± 28.55	82.14 ± 25.14	81.89 ± 27.46	82.50 ± 26.73	84.03 ± 34.01	<0.01
eGFR (mL/min)	88.76 ± 20.83	89.80 ± 21.32	89.69 ± 19.83	88.72 ± 20.03	86.85 ± 21.91	<0.01
The number of FPG measurements						<0.01
2	41,828 (42.61)	13,518 (54.70)	8992 (36.48)	8779 (36.21)	10,539 (42.92)	
3	56,333 (57.39)	11,195 (45.30)	15,659 (63.52)	15,464 (63.79)	14,015 (57.08)	
FPG variability						
^†^CV	8.14 (4.56, 13.20)	2.69 (1.50, 3.70)	6.39 (5.48, 7.40)	10.44 (9.16, 11.92)	18.25 (15.13, 24.04)	<0.01
^†^ARV	0.54 (0.30, 0.94)	0.18 (0.10, 0.25)	0.42 (0.35, 0.52)	0.71 (0.59, 0.86)	1.39 (1.09, 2.02)	<0.01
^†^VIM	0.43 (0.24, 0.71)	0.14 (0.08, 0.19)	0.33 (0.28, 0.38)	0.55 (0.49, 0.62)	1.03 (0.84, 1.53)	<0.01

Note: Q1: standard deviation of FPG < 0.24; Q2: 0.24 ≤ standard deviation of FPG < 0.43; Q3: 0.43 ≤ standard deviation of FPG < 0.77; Q4: standard deviation of FPG ≥ 0.77. Low-income level: income ≥ 800 renminbi/month. Continuous variables are presented as mean ± SD, and categorical variables are presented as percentage. Abbreviations: ARV: average real variability; BMI: body mass index; CKD: chronic kidney disease; CV: coefficient of variance; DBP: diastolic blood pressure; eGFR: estimated glomerular filtration rate; FPG: fasting plasma glucose; HDL-C: high-density lipoprotein cholesterol; LDL-C: low-density lipoprotein cholesterol; RHR: resting heart rate; SBP: systolic blood pressure; SD: standard deviation; Scr: serum creatinine levels; TC: total cholesterol; TG: triglyceride; VIM: variability independent of mean. ^∗^The eGFR was calculated using the Chronic Kidney Disease Epidemiology Collaboration equation. Chronic kidney disease (CKD) was defined as an eGFR (calculated using the modification of diet in renal disease formula) under 60 mL/min at the baseline of health examination. ^†^Triglyceride levels and the SD, CV, ARV, and VIM of fasting plasma glucose levels are presented as median (interquartile range).

**Table 2:** Risks of total digestive cancers according to quartiles of FPG variability assessed by SD.

Variable	HR (95% CI) according to quartiles of variability of FPG			
	Quartile 1	Quartile 2	Quartile 3	Quartile 4
Event, *n* (%)	213 (0.86)	244 (0.99)	299 (1.23)	347 (1.41)
IR^∗^	0.92	1.07	1.33	1.51
Cox regression models				
Model 1	1 (reference)	1.342 (1.117-1.614)	1.557 (1.305-1.856)	1.425 (1.201-1.692)
*P* value	<0.0001^†^	0.0017	<0.0001	<0.0001
Model 2	1 (reference)	1.342 (1.117-1.613)	1.554 (1.303-1.853)	1.391 (1.163-1.663)
*P* value	<0.0001^†^	0.0017	<0.0001	0.0003
Model 3	1 (reference)	1.341 (1.116-1.612)	1.551 (1.301-1.850)	1.387 (1.16-1.659)
*P* value	<0.0001^†^	0.0018	<0.0001	0.0003

^∗^IR (incidence rate) presented as per 1000 person-years. ^†^*P* for trend. Model 1: adjusted for age and sex. Model 2: model 1 + LDL-C, baseline FPG, antihypertensive drugs, hypoglycemic drugs, hypertension, and diabetes mellitus. Model 3: model 2 + BMI, current smoking, current drinking, physical exercise, and family history of cancer. Abbreviations: CI: confidence interval; FPG: fasting plasma glucose; HR: hazard ratio; SD: standard deviation.

**Table 3:** Risks of site-specific digestive cancer according to quartiles of FPG variability assessed by SD.

Variables	HR (95% CI) according to quartiles of variability of FPG			
	Quartile 1	Quartile 2	Quartile 3	Quartile 4
Gastric and esophageal				
Events, *n* (%)	50 (0.2)	47 (0.19)	59 (0.24)	69 (0.28)
IR^∗^	0.2	0.19	0.24	0.28
Cox regression models				
Model 1	1 (reference)	1.109 (0.744-1.652)	1.310 (0.898-1.911)	1.187 (0.824-1.711)
*P* value	0.5518^†^	0.6118	0.1603	0.3568
Model 2	1 (reference)	1.107 (0.743-1.649)	1.310 (0.898-1.911)	1.105 (0.785-1.686)
*P* value	0.5662^†^	0.6169	0.1610	0.4726
Model 3	1 (reference)	1.100 (0.738-1.639)	1.300 (0.891-1.897)	1.139 (0.776-1.670)
*P* value	0.5877^†^	0.6403	0.1732	0.5062
Liver				
Events, *n* (%)	46 (0.19)	55 (0.22)	68 (0.28)	74 (0.3)
IR^∗^	0.19	0.22	0.28	0.30
Cox regression models				
Model 1	1 (reference)	1.412 (0.954-2.091)	1.689 (1.161-2.456)	1.470 (1.016-2.127)
*P* value	0.0517^†^	0.0843	0.0062	0.0407
Model 2	1 (reference)	1.411 (0.953-2.089)	1.680 (1.155-2.445)	1.441 (0.984-2.111)
*P* value	0.0581^†^	0.0853	0.0067	0.0606
Model 3	1 (reference)	1.407 (0.95-2.082)	1.682 (1.156-2.449)	1.427 (0.973-2.092)
*P* value	0.0585^†^	0.0881	0.0066	0.0686
Pancreatic				
Events, *n* (%)	11 (0.04)	26 (0.11)	17 (0.07)	29 (0.12)
IR^∗^	0.04	0.11	0.07	0.12
Cox regression models				
Model 1	1 (reference)	2.741 (1.354-5.551)	1.667 (0.78-3.562)	2.207 (1.101-4.425)
*P* value	0.0335^†^	0.0051	0.1873	0.0257
Model 2	1 (reference)	2.741 (1.354-5.552)	1.660 (0.776-3.549)	2.090 (1.017-4.294)
*P* value	0.0374^†^	0.0051	0.1911	0.0449
Model 3	1 (reference)	2.743 (1.354-5.556)	1.674 (0.783-3.579)	2.105 (1.024-4.329)
*P* value	0.0376^†^	0.0051	0.1842	0.0430
Colorectal				
Events, *n* (%)	82 (0.33)	82 (0.33)	120 (0.49)	135 (0.55)
IR^∗^	0.33	0.33	0.49	0.55
Cox regression models				
Model 1	1 (reference)	1.165 (0.858-1.583)	1.603 (1.210-2.124)	1.433 (1.088-1.887)
*P* value	0.0047^†^	0.3285	0.0010	0.0105
Model 2	1 (reference)	1.166 (0.858-1.584)	1.612 (1.217-2.136)	1.443 (1.081-1.926)
*P* value	0.0045^†^	0.3269	0.0009	0.0128
Model 3	1 (reference)	1.164 (0.856-1.581)	1.600 (1.207-2.121)	1.432 (1.073-1.912)
*P* value	0.0056^†^	0.3324	0.0011	0.0149

^∗^IR (incidence rate) presented as per 1000 person-years. ^†^*P* for trend. Model 1: adjusted for age and sex. Model 2: model 1 + LDL-C, baseline FPG, antihypertensive drugs, hypoglycemic drugs, hypertension, and diabetes mellitus. Model 3: model 2 + BMI, current smoking, current drinking, physical exercise, and family history of cancer. Abbreviations: CI: confidence interval; FPG: fasting plasma glucose; HR: hazard ratio; SD: standard deviation.

## Data Availability

The data in this study could be made available upon reasonable request to the corresponding authors.

## Abbreviations

ARV: Average successive variability
BMI: Body mass index
CKD: Chronic kidney disease
CV: Coefficient of variation
eGFR: Estimated glomerular filtration rate
FPG: Fasting plasma glucose
ICD: International Classification of Diseases
SD: Standard deviation
T2DM: Type 2 diabetes mellitus
VIM: Variability independent of the mean.

## Supplementary Materials

Supplementary 1 Figure S1: proportion of the major types of incident digestive cancers in the Kailuan study during follow-up. Table S1: baseline characteristics according to quartiles of FPG variability assessed by CV. Table S2: baseline characteristics according to quartiles of FPG variability assessed by VIM. Table S3: baseline characteristics according to quartiles of FPG variability assessed by ARV. Table S4: risks of total digestive cancers according to quartiles of FPG variability assessed by CV. Table S5: risks of total digestive cancers according to quartiles of FPG variability assessed by VIM. Table S6: risks of total digestive cancers according to quartiles of FPG variability assessed by ARV. Table S7: risks of site-specific digestive cancer according to quartiles of FPG variability assessed by CV. Table S8: risks of site-specific digestive cancer according to quartiles of FPG variability assessed by VIM. Table S9: risks of site-specific digestive cancer according to quartiles of FPG variability assessed by ARV. Table S10: subgroup analyses according to quartiles of FPG variability assessed by SD. Table S11: subgroup analyses according to quartiles of FPG variability assessed by CV. Table S12: subgroup analyses according to quartiles of FPG variability assessed by VIM. Table S13: subgroup analyses according to quartiles of FPG variability assessed by ARV.

Supplementary 2 STROBE checklist.

## References

[1] Sung H., Ferlay J., Siegel R. L. (2021). Global cancer statistics 2020: GLOBOCAN estimates of incidence and mortality worldwide for 36 cancers in 185 countries. *CA: a Cancer Journal for Clinicians*.

[2] Feng R. M., Zong Y. N., Cao S. M., Xu R. H. (2019). Current cancer situation in China: good or bad news from the 2018 global cancer statistics?. *Cancer Commun (Lond)*.

[3] Egnell M., Fassier P., Lecuyer L. (2017). Antioxidant intake from diet and supplements and risk of digestive cancers in middle-aged adults: results from the prospective NutriNet-Santé cohort. *The British Journal of Nutrition*.

[4] Xie W. Q., Wang X. F. (2017). MiR-146a rs2910164 polymorphism increases the risk of digestive system cancer: a meta-analysis. *Clinics and Research in Hepatology and Gastroenterology*.

[5] Tsilidis K. K., Kasimis J. C., Lopez D. S., Ntzani E. E., Ioannidis J. P. (2014). Type 2 diabetes and cancer: umbrella review of meta-analyses of observational studies. *BMJ*.

[6] Liao W. C., Tu Y. K., Wu M. S., Lin J. T., Wang H. P., Chien K. L. (2015). Blood glucose concentration and risk of pancreatic cancer: systematic review and dose-response meta-analysis. *BMJ*.

[7] Shi J., Xiong L., Li J. (2015). A linear dose-response relationship between fasting plasma glucose and colorectal cancer risk: systematic review and meta-analysis. *Scientific Reports*.

[8] Han H., Zhang T., Jin Z. (2017). Blood glucose concentration and risk of liver cancer: systematic review and meta-analysis of prospective studies. *Oncotarget*.

[9] Lee S., Zhou J., Wong W. T. (2021). Glycemic and lipid variability for predicting complications and mortality in diabetes mellitus using machine learning. *BMC Endocrine Disorders*.

[10] Frontoni S., Di Bartolo P., Avogaro A., Bosi E., Paolisso G., Ceriello A. (2013). Glucose variability: an emerging target for the treatment of diabetes mellitus. *Diabetes Research and Clinical Practice*.

[11] Lee S., Liu T., Zhou J., Zhang Q., Wong W. T., Tse G. (2021). Predictions of diabetes complications and mortality using hba1c variability: a 10-year observational cohort study. *Acta Diabetologica*.

[12] Monnier L., Mas E., Ginet C. (2006). Activation of oxidative stress by acute glucose fluctuations compared with sustained chronic hyperglycemia in patients with type 2 diabetes. *JAMA*.

[13] Masoudkabir F., Sarrafzadegan N. (2020). The interplay of endothelial dysfunction, cardiovascular disease, and cancer: what we should know beyond inflammation and oxidative stress. *European Journal of Preventive Cardiology*.

[14] Lin C. C., Li C. I., Liu C. S. (2012). Annual fasting plasma glucose variation increases risk of cancer incidence and mortality in patients with type 2 diabetes: the Taichung Diabetes Study. *Endocrine-Related Cancer*.

[15] Wu M., Lu J., Yang Z. (2021). Longitudinal changes in fasting plasma glucose are associated with risk of cancer mortality: a Chinese cohort study. *Cancer Medicine*.

[16] Feng X., Wang G., Li N. (2017). The association between fasting blood glucose and the risk of primary liver cancer in Chinese males: a population-based prospective study. *British Journal of Cancer*.

[17] Muntner P., Whittle J., Lynch A. I. (2015). Visit-to-visit variability of blood pressure and coronary heart disease, stroke, heart failure, and mortality: a cohort study. *Annals of Internal Medicine*.

[18] Zhao X., Wang N., Sun Y. (2020). Screen-detected gallstone disease and risk of liver and pancreatic cancer: the Kailuan cohort study. *Liver International*.

[19] Giovannucci E., Harlan D. M., Archer M. C. (2010). Diabetes and cancer: a consensus report. *CA: a Cancer Journal for Clinicians*.

[20] Yamagata H., Kiyohara Y., Nakamura S. (2005). Impact of fasting plasma glucose levels on gastric cancer incidence in a general Japanese population: the Hisayama study. *Diabetes Care*.

[21] Ceriello A., Monnier L., Owens D. (2019). Glycaemic variability in diabetes: clinical and therapeutic implications. *The Lancet Diabetes and Endocrinology*.

[22] Bi J., Song L., Wang L. (2021). Visit-to-visit fasting blood glucose variability and lifetime risk of cardiovascular disease: a prospective study. *Cardiovascular Diabetology*.

[23] Gerbaud E., Darier R., Montaudon M. (2019). Glycemic variability is a powerful independent predictive factor of midterm major adverse cardiac events in patients with diabetes with acute coronary syndrome. *Diabetes Care*.

[24] Lee S., Jeevaratnam K., Liu T. (2021). Risk stratification of cardiac arrhythmias and sudden cardiac death in type 2 diabetes mellitus patients receiving insulin therapy: a population-based cohort study. *Clinical Cardiology*.

[25] Jun H., Lee J., Lee H. A. (2022). Fasting blood glucose variability and unfavorable trajectory patterns are associated with the risk of colorectal cancer. *Gut Liver*.

[26] Hong S. H., Noh E., Kim J. (2020). Fasting plasma glucose variability and gastric cancer risk in individuals without diabetes mellitus: a nationwide population-based cohort study. *Clinical and Translational Gastroenterology*.

[27] Klimontov V. V., Saik O. V., Korbut A. I. (2021). Glucose variability: how does it work?. *International Journal of Molecular Sciences*.

[28] D'Souza L. C., Mishra S., Chakraborty A., Shekher A., Sharma A., Gupta S. C. (2020). Oxidative stress and cancer development: are noncoding RNAs the missing links?. *Antioxidants & Redox Signaling*.

[29] Greten F. R., Grivennikov S. I. (2019). Inflammation and cancer: triggers, mechanisms, and consequences. *Immunity*.

[30] Tay J., Thompson C. H., Brinkworth G. D. (2015). Glycemic variability: assessing glycemia differently and the implications for dietary management of diabetes. *Annual Review of Nutrition*.

[31] Key T. J., Bradbury K. E., Perez-Cornago A., Sinha R., Tsilidis K. K., Tsugane S. (2020). Diet, nutrition, and cancer risk: what do we know and what is the way forward?. *BMJ*.

[32] Luo Y., Ma J., Lu W. (2020). The significance of mitochondrial dysfunction in cancer. *International Journal of Molecular Sciences*.

[33] Tahara T., Arisawa T. (2012). Potential usefulness of DNA methylation as a risk marker for digestive cancer associated with inflammation. *Expert Review of Molecular Diagnostics*.

[34] De Maria N., Manno M., Villa E. (2002). Sex hormones and liver cancer. *Molecular and Cellular Endocrinology*.

[35] Murphy N., Strickler H. D., Stanczyk F. Z. (2015). A prospective evaluation of endogenous sex hormone levels and colorectal cancer risk in postmenopausal women. *Journal of the National Cancer Institute*.

[36] Kamarudin M. N. A., Sarker M. M. R., Zhou J. R., Parhar I. (2019). Metformin in colorectal cancer: molecular mechanism, preclinical and clinical aspects. *Journal of Experimental & Clinical Cancer Research*.

[37] Okumura T. (2010). Mechanisms by which thiazolidinediones induce anti-cancer effects in cancers in digestive organs. *Journal of Gastroenterology*.

[38] Kaji K., Nishimura N., Seki K. (2018). Sodium glucose cotransporter 2 inhibitor canagliflozin attenuates liver cancer cell growth and angiogenic activity by inhibiting glucose uptake. *International Journal of Cancer*.

