# Supplementary material for: Association of Visit-to-Visit Variability in Fasting Plasma Glucose with Digestive Cancer Risk
Source: Oxid Med Cell Longev. 2022 Jul 13;2022:4530894. doi: 10.1155/2022/4530894 (PMC9301759; doi:10.1155/2022/4530894)
Supplement: Supplementary 1 — Figure S1: proportion of the major types of incident digestive cancers in the Kailuan study during follow-up. Table S1: baseline characteristics according to quartiles of FPG variability assessed by CV. Table S2: baseline characteristics according to quartiles of FPG variability assessed by VIM. Table S3: baseline characteristics according to quartiles of FPG variability assessed by ARV. Table S4: risks of total digestive cancers according to quartiles of FPG variability assessed by CV. Table S5: risks of total digestive cancers according to quartiles of FPG variability assessed by VIM. Table S6: risks of total digestive cancers according to quartiles of FPG variability assessed by ARV. Table S7: risks of site-specific digestive cancer according to quartiles of FPG variability assessed by CV. Table S8: risks of site-specific digestive cancer according to quartiles of FPG variability assessed by VIM. Table S9: risks of site-specific digestive cancer according to quartiles of FPG variability assessed by ARV. Table S10: subgroup analyses according to quartiles of FPG variability assessed by SD. Table S11: subgroup analyses according to quartiles of FPG variability assessed by CV. Table S12: subgroup analyses according to quartiles of FPG variability assessed by VIM. Table S13: subgroup analyses according to quartiles of FPG variability assessed by ARV. [file 4530894.f1.docx]

**- Supplement materials –**

**Supplementary figure and tables**

**Figure S1 Proportion of the major types of incident digestive cancers in the Kailuan study during follow-up.**

**Table S1. Baseline characteristics according to quartiles of FPG variability assessed by CV**

**Table S2. Baseline characteristics according to quartiles of FPG variability assessed by VIM**

**Table S3. Baseline characteristics according to quartiles of FPG variability assessed by ARV**

**Table S4. Risks of total digestive cancers according to quartiles of FPG variability assessed by CV**

**Table S5. Risks of total digestive cancers according to quartiles of FPG variability assessed by VIM**

**Table S6. Risks of total digestive cancers according to quartiles of FPG variability assessed by ARV**

**Table S7. Risks of site-specific digestive cancer according to quartiles of FPG variability assessed by CV**

**Table S8. Risks of site-specific digestive cancer according to quartiles of FPG variability assessed by VIM**

**Table S9. Risks of site-specific digestive cancer according to quartiles of FPG variability assessed by ARV**

**Table S10. Subgroup analyses according to quartiles of FPG variability assessed by SD**

**Table S11. Subgroup analyses according to quartiles of FPG variability assessed by CV**

**Table S12. Subgroup analyses according to quartiles of FPG variability assessed by VIM**

**Table S13. Subgroup analyses according to quartiles of FPG variability assessed by ARV**

**
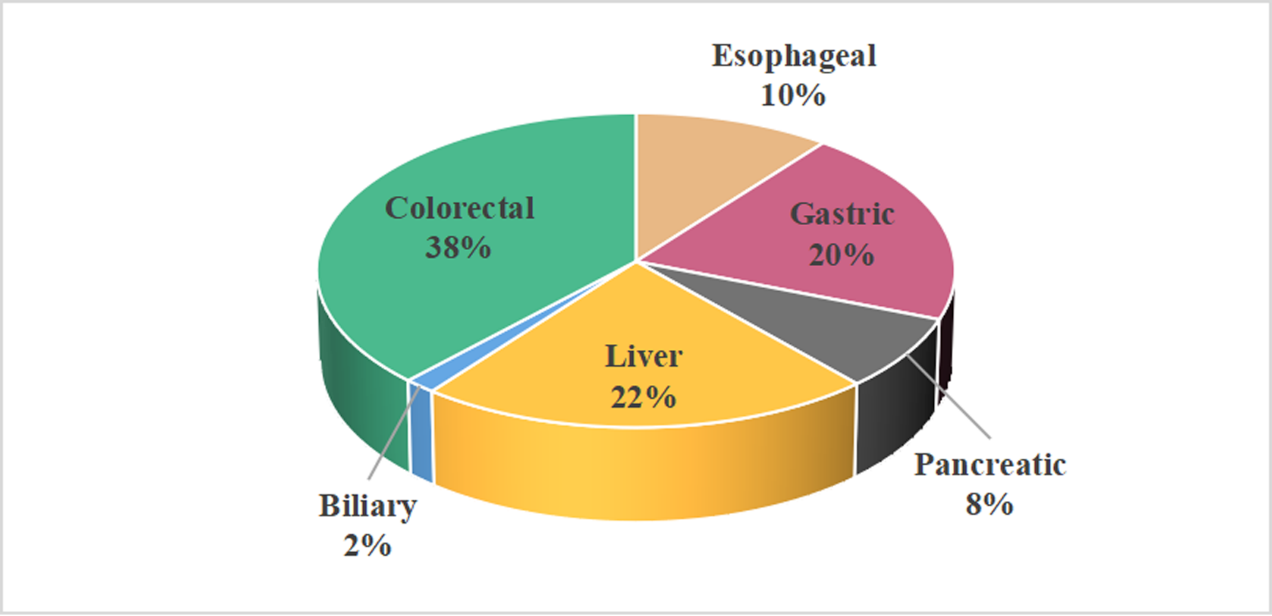
**

**Figure S1** Proportion of the major types of incident digestive cancers in the Kailuan study during follow-up.

**Table S1. Baseline characteristics according to quartiles of FPG variability assessed by CV**

| **Variables** | **Total**  **N=98161** | **Quartile 1**  **N=24548** | **Quartile 2**  **N=24521** | **Quartile 3**  **N=24554** | **Quartile 4**  **N=24538** | **P**  **value** |
| --- | --- | --- | --- | --- | --- | --- |
| Age, y | 53.62±12.35 | 52.25±12.36 | 52.64±12.31 | 53.80±12.42 | 55.79±11.99 | <0.01 |
| Male sex, n (%) | 77492 (78.94) | 19152 (78.02) | 18987 (77.43) | 19501 (79.42) | 19852 (80.90) | <0.01 |
| Comorbidities |  |  |  |  |  |  |
| Hypertension, n (%) | 15471 (15.76) | 3309 (13.48) | 3551 (14.48) | 3895 (15.86) | 4716 (19.22) | <0.01 |
| Diabetes mellitus, n (%) | 4383 (4.47) | 463 (1.89) | 545 (2.22) | 805 (3.28) | 2570 (10.47) | <0.01 |
| Dyslipidemia, n (%) | 4638 (4.72) | 952 (3.88) | 1088 (4.44) | 1225 (4.99) | 1373 (5.60) | <0.01 |
| *CKD, eGFR <60 mL/min, n (%) | 8354 (8.51) | 1988 (8.10) | 1835 (7.48) | 2059 (8.39) | 2472 (10.07) | <0.01 |
| Family history of cancer | 3919 (3.99) | 908 (3.70) | 961 (3.92) | 972 (3.96) | 1078 (4.39) | <0.01 |
| Medications |  |  |  |  |  |  |
| Antihypertensive agents, n (%) | 14387 (14.66) | 3020 (12.30) | 3289 (13.41) | 3626 (14.77) | 4452 (18.14) | <0.01 |
| Hypoglycemic agents, n (%) | 3799 (3.87) | 396 (1.61) | 459 (1.87) | 693 (2.82) | 2251 (9.17) | <0.01 |
| Lipid-lowers agents, n (%) | 978 (1.00) | 164 (0.67) | 202 (0.82) | 256 (1.04) | 356 (1.45) | <0.01 |
| Lifestyle |  |  |  |  |  |  |
| Current smoker, n (%) | 33502 (34.13) | 8244 (33.58) | 8197 (33.43) | 8543 (34.79) | 8518 (34.71) | <0.01 |
| Current alcohol use, n (%) | 34711 (35.36) | 8848 (36.04) | 8624 (35.17) | 8802 (35.85) | 8437 (34.38) | <0.01 |
| Regular exercise, n (%) | 68808 (70.10) | 17497 (71.28) | 17172 (70.03) | 17103 (69.65) | 17036 (69.43) | <0.01 |
| Low-income level, n (%) | 46846 (47.72) | 11259 (45.87) | 11920 (48.61) | 11987 (48.82) | 11680 (47.60) | <0.01 |
| Health examination |  |  |  |  |  |  |
| Body weight, kg | 71.16±11.29 | 70.82±11.26 | 70.96±11.34 | 71.10±11.23 | 71.76±11.30 | <0.01 |
| BMI, kg/m^2^ | 25.09±3.40 | 24.93±3.36 | 25.00±3.35 | 25.06±3.39 | 25.36±3.47 | <0.01 |
| RHR, bpm | 73.63±10.50 | 73.12±10.08 | 73.15±10.13 | 73.47±10.42 | 74.79±11.25 | <0.01 |
| SBP, mmHg | 132.03±20.14 | 130.42±20.15 | 130.65±19.73 | 132.20±20.03 | 134.84±20.33 | <0.01 |
| DBP, mmHg | 84.72±11.17 | 84.19±11.15 | 84.27±11.11 | 84.79±11.21 | 85.61±11.14 | <0.01 |
| FPG, mmol/L | 5.69±1.85 | 5.34±0.96 | 5.39±1.03 | 5.55±1.27 | 6.48±3.05 | <0.01 |
| TC, mmol/L | 5.01±1.38 | 4.95±1.19 | 4.98±1.25 | 5.02±1.64 | 5.08±1.38 | <0.01 |
| †TG, mmol/L | 1.29 (0.92,1.92) | 1.25 (0.88,1.84) | 1.25 (0.90,1.86) | 1.29 (0.92,1.90) | 1.37 (0.99,2.07) | <0.01 |
| LDL-C, mmol/L | 2.62±1.07 | 2.62±0.91 | 2.64±1.17 | 2.62±1.11 | 2.60±1.10 | <0.01 |
| HDL-C, mmol/L | 1.54±0.53 | 1.53±0.51 | 1.54±0.51 | 1.55±0.52 | 1.55±0.58 | <0.01 |
| Scr, μmol/L | 82.64±28.55 | 82.26±25.07 | 81.84±26.83 | 82.61±27.98 | 83.85±33.56 | <0.01 |
| eGFR, mL/min | 88.76±20.83 | 89.65±21.27 | 89.70±19.71 | 88.69±20.43 | 87.02±21.72 | <0.01 |
| The number of FPG measurements |  |  |  |  |  | <0.01 |
| 2 | 41828 (42.61) | 13575 (55.30) | 8927 (36.41) | 8827 (35.95) | 10499 (42.79) |  |
| 3 | 56333 (57.39) | 10973 (44.70) | 15594 (63.59) | 15727 (64.05) | 14039 (57.21) |  |
| FPG Variability |  |  |  |  |  |  |
| †SD | 0.43 (0.24,0.71) | 0.14 (0.08,0.19) | 0.33 (0.28,0.38) | 0.54 (0.47,0.62) | 1.01 (0.81,1.52) | <0.01 |
| †ARV | 0.54 (0.30,0.94) | 0.18 (0.10,0.25) | 0.42 (0.35,0.52) | 0.70 (0.57,0.86) | 1.35 (1.04,2.00) | <0.01 |
| †VIM | 0.43 (0.24,0.71) | 0.14 (0.08,0.19) | 0.33 (0.28,0.38) | 0.54 (0.47,0.62) | 1.01 (0.81,1.52) | <0.01 |

Note: Q1, the coefficient of variability of FPG < 4.56 mmol/L; Q2, 4.56 mmol/L ≤ the coefficient of variability of FPG < 8.14 mmol/L; Q3, 8.14 mmol/L ≤ the coefficient of variability of FPG < 13.20 mmol/L; Q4, the coefficient of variability of FPG ≥ 13.20 mmol/L.

Low-income level: income≥800 Renminbi/month.

Continuous variables are presented as mean±SD, and categorical variables are presented as percentage.

Abbreviations: CKD, chronic kidney disease; eGFR, estimated glomerular filtration rate; Scr, serum creatinine levels; BMI, body mass index; DBP, diastolic blood pressure; SBP, systolic blood pressure; FPG, fasting blood glucose; HDL-C, high-density lipoprotein cholesterol; LDL-C, low-density lipoprotein cholesterol; RHR, resting heart rate; TC, total cholesterol; TG, triglyceride; VIM, variability independent of mean; SD, the standard deviation; ARV, average real variability.

* The eGFR was calculated using the Chronic Kidney Disease Epidemiology Collaboration equation. Chronic kidney disease; CKD was defined as an eGFR (calculated using the Modification of Diet in Renal Disease formula) under 60 mL/min at the baseline of health examination.

† Triglyceride levels and the SD, CV, ARV, VIM of fasting plasma glucose levels are presented as median (interquartile range).

**Table S2. Baseline characteristics according to quartiles of FPG variability assessed by VIM**

| **Variables** | **Total**  **N=98161** | **Quartile 1**  **N=24721** | **Quartile 2**  **N=24646** | **Quartile 3**  **N=27208** | **Quartile 4**  **N=21586** | **P**  **value** |
| --- | --- | --- | --- | --- | --- | --- |
| Age, y | 53.62±12.35 | 51.97±12.46 | 52.50±12.41 | 53.93±12.38 | 56.40±11.57 | <0.01 |
| Male sex, n (%) | 77492 (78.94) | 19086 (77.21) | 18964 (76.95) | 21692 (79.73) | 17750 (82.23) | <0.01 |
| Comorbidities |  |  |  |  |  |  |
| Hypertension, n (%) | 15471 (15.76) | 3203 (12.96) | 3432 (13.93) | 4306 (15.83) | 4530 (20.99) | <0.01 |
| Diabetes mellitus, n (%) | 4383 (4.47) | 327 (1.32) | 324 (1.31) | 643 (2.36) | 3089 (14.31) | <0.01 |
| Dyslipidemia, n (%) | 4638 (4.72) | 933 (3.77) | 1031 (4.18) | 1328 (4.88) | 1346 (6.24) | <0.01 |
| *CKD, eGFR <60 mL/min, n (%) | 8354 (8.51) | 1975 (7.99) | 1890 (7.67) | 2220 (8.16) | 2269 (10.51) | <0.01 |
| Family history of cancer | 3919 (3.99) | 898 (3.63) | 980 (3.98) | 1104 (4.06) | 937 (4.34) | <0.01 |
| Medications |  |  |  |  |  |  |
| Antihypertensive agents, n (%) | 14387 (14.66) | 2917 (11.80) | 3174 (12.88) | 4020 (14.78) | 4276 (19.81) | <0.01 |
| Hypoglycemic agents, n (%) | 3799 (3.87) | 273 (1.10) | 264 (1.07) | 553 (2.03) | 2709 (12.55) | <0.01 |
| Lipid-lowers agents, n (%) | 978 (1.00) | 155 (0.63) | 203 (0.82) | 264 (0.97) | 356 (1.65) | <0.01 |
| Lifestyle |  |  |  |  |  |  |
| Current smoker, n (%) | 33502 (34.13) | 8293 (33.55) | 8130 (32.99) | 9591 (35.25) | 7488 (34.69) | <0.01 |
| Current alcohol use, n (%) | 34711 (35.36) | 8823 (35.69) | 8557 (34.72) | 9867 (36.27) | 7464 (34.58) | 0.14 |
| Regular exercise, n (%) | 68808 (70.10) | 17617 (71.26) | 17265 (70.05) | 18843 (69.26) | 15083 (69.87) | 0.01 |
| Low-income level, n (%) | 51315 (52.28) | 13404 (54.22) | 12715 (51.59) | 13786 (50.67) | 11410 (52.86) | <0.01 |
| Health examination |  |  |  |  |  |  |
| Body weight, kg | 71.16±11.29 | 70.54±11.29 | 70.81±11.34 | 71.12±11.19 | 72.32±11.27 | <0.01 |
| BMI, kg/m^2^ | 25.09±3.40 | 24.84±3.36 | 24.96±3.35 | 25.06±3.37 | 25.54±3.48 | <0.01 |
| RHR, bpm | 73.63±10.50 | 72.97±10.02 | 72.90±10.06 | 73.46±10.43 | 75.44±11.38 | <0.01 |
| SBP, mmHg | 132.03±20.14 | 129.79±20.03 | 130.13±19.58 | 132.34±19.92 | 136.36±20.44 | <0.01 |
| DBP, mmHg | 84.72±11.17 | 83.94±11.15 | 84.01±11.06 | 84.93±11.15 | 86.15±11.18 | <0.01 |
| FPG, mmol/L | 5.69±1.85 | 5.22±0.73 | 5.27±0.76 | 5.47±0.98 | 6.99±3.31 | <0.01 |
| TC, mmol/L | 5.01±1.38 | 4.93±1.12 | 4.96±1.37 | 5.02±1.53 | 5.13±1.44 | <0.01 |
| †TG, mmol/L | 1.29 (0.92,1.92) | 1.23 (0.87,1.81) | 1.24 (0.89,1.83) | 1.30 (0.93,1.90) | 1.41 (1.01,2.16) | <0.01 |
| LDL-C, mmol/L | 2.62±1.07 | 2.61±0.91 | 2.62±1.07 | 2.62±1.17 | 2.64±1.14 | 0.01 |
| HDL-C, mmol/L | 1.54±0.53 | 1.53±0.52 | 1.54±0.50 | 1.55±0.52 | 1.54±0.58 | <0.01 |
| Scr, μmol/L | 82.64±28.55 | 82.14±25.14 | 81.89±27.46 | 82.42±26.75 | 84.34±34.86 | <0.01 |
| eGFR, mL/min | 88.76±20.83 | 89.80±21.32 | 89.69±19.83 | 88.76±20.03 | 86.54±22.14 | <0.01 |
| The number of FPG measurements |  |  |  |  |  | <0.01 |
| 2 | 41828 (42.61) | 13521 (54.69) | 8989 (36.47) | 9799 (36.02) | 9519 (44.10) |  |
| 3 | 56333 (57.39) | 11200 (45.31) | 15657 (63.53) | 17409 (63.98) | 12067 (55.90) |  |
| FPG Variability |  |  |  |  |  |  |
| †SD | 0.43 (0.24,0.71) | 0.14 (0.08,0.19) | 0.33 (0.28,0.38) | 0.57 (0.49,0.65) | 1.11 (0.90,1.66) | <0.01 |
| †ARV | 0.54 (0.30,0.94) | 0.18 (0.10,0.25) | 0.42 (0.35,0.52) | 0.73 (0.61,0.89) | 1.49 (1.17,2.20) | <0.01 |
| †CV | 8.14 (4.56,13.20) | 2.69 (1.50,3.70) | 6.39 (5.48,7.40) | 10.71(9.29,12.44) | 19.25 (16.04,25.23) | <0.01 |

Note: Q1, the variability independent of the mean of FPG < 0.24; Q2, 0.24 ≤ the variability independent of the mean of FPG < 0.43; Q3, 0.43 ≤ the variability independent of the mean of FPG < 0.77; Q4, the variability independent of the mean of FPG ≥ 0.77.

Low-income level: income≥800 Renminbi/month.

Continuous variables are presented as mean±SD, and categorical variables are presented as percentage.

Abbreviations: ARV, average real variability; BMI, body mass index; CKD, chronic kidney disease; CV, coefficient of variance; DBP, diastolic blood pressure; eGFR, estimated glomerular filtration rate; FPG, fasting plasma glucose; HDL-C, high-density lipoprotein cholesterol; LDL-C, low-density lipoprotein cholesterol; RHR, resting heart rate; SBP, systolic blood pressure; SD, standard deviation; Scr, serum creatinine levels; TC, total cholesterol; TG, triglyceride; VIM, variability independent of mean.

* The eGFR was calculated using the Chronic Kidney Disease Epidemiology Collaboration equation. Chronic kidney disease; CKD was defined as an eGFR (calculated using the Modification of Diet in Renal Disease formula) under 60 mL/min at the baseline of health examination.

† Triglyceride levels and the SD, CV, ARV, VIM of fasting plasma glucose levels are presented as median (interquartile range).

**Table S3. Baseline characteristics according to quartiles of FPG variability assessed by ARV**

| **Variables** | **Total**  **N=98161** | **Quartile 1**  **N=24704** | **Quartile 2**  **N=24197** | **Quartile 3**  **N=24840** | **Quartile 4**  **N=24420** | **P**  **value** |
| --- | --- | --- | --- | --- | --- | --- |
| Age, y | 53.62±12.35 | 51.96±12.45 | 52.55±12.40 | 54.00±12.31 | 55.97±11.83 | <0.01 |
| Male sex, n (%) | 77492 (78.94) | 18985 (76.85) | 18717 (77.35) | 19787 (79.66) | 20003 (81.91) | <0.01 |
| Comorbidities |  |  |  |  |  |  |
| Hypertension, n (%) | 15471 (15.76) | 3220 (13.03) | 3392 (14.02) | 3911 (15.74) | 4948 (20.26) | <0.01 |
| Diabetes mellitus, n (%) | 4383 (4.47) | 312 (1.26) | 349 (1.44) | 594 (2.39) | 3128 (12.81) | <0.01 |
| Dyslipidemia, n (%) | 4638 (4.72) | 956 (3.87) | 1075 (4.44) | 1159 (4.67) | 1448 (5.93) | <0.01 |
| *CKD, eGFR <60 mL/min, n (%) | 8354 (8.51) | 1867 (7.56) | 1889 (7.81) | 2028 (8.16) | 2570 (10.52) | <0.01 |
| Family history of cancer | 3919 (3.99) | 893 (3.61) | 978 (4.04) | 1016 (4.09) | 1032 (4.23) | <0.01 |
| Medications |  |  |  |  |  |  |
| Antihypertensive agents, n (%) | 14387 (14.66) | 2952 (11.95) | 3116 (12.88) | 3648 (14.69) | 4671 (19.13) | <0.01 |
| Hypoglycemic agents, n (%) | 3799 (3.87) | 258 (1.04) | 298 (1.23) | 505 (2.03) | 2738 (11.21) | <0.01 |
| Lipid-lowers agents, n (%) | 978 (1.00) | 164 (0.66) | 196 (0.81) | 236 (0.95) | 382 (1.56) | <0.01 |
| Lifestyle |  |  |  |  |  |  |
| Current smoker, n (%) | 33502 (34.13) | 8250 (33.40) | 8113 (33.53) | 8616 (34.69) | 8523 (34.90) | <0.01 |
| Current alcohol use, n (%) | 34711 (35.36) | 8783 (35.55) | 8461 (34.97) | 8903 (35.84) | 8564 (35.07) | 0.14 |
| Regular exercise, n (%) | 68808 (70.10) | 17497 (70.83) | 16867 (69.71) | 17299 (69.64) | 17145 (70.21) | 0.01 |
| Low-income level, n (%) | 51315 (52.28) | 13152 (53.24) | 12399 (51.24) | 12642 (50.89) | 13122 (53.73) | <0.01 |
| Health examination |  |  |  |  |  |  |
| Body weight, kg | 71.16±11.29 | 70.53±11.29 | 70.89±11.30 | 71.15±11.20 | 72.07±11.29 | <0.01 |
| BMI, kg/m2 | 25.09±3.40 | 24.85±3.35 | 24.99±3.37 | 25.07±3.37 | 25.45±3.46 | <0.01 |
| RHR, bpm | 73.63±10.50 | 72.89±9.99 | 72.99±10.06 | 73.46±10.45 | 75.20±11.30 | <0.01 |
| SBP, mmHg | 132.03±20.14 | 129.63±19.89 | 130.25±19.61 | 132.41±19.94 | 135.82±20.51 | <0.01 |
| DBP, mmHg | 84.72±11.17 | 83.85±11.08 | 84.17±11.02 | 84.91±11.24 | 85.94±11.22 | <0.01 |
| FPG, mmol/L | 5.69±1.85 | 5.22±0.72 | 5.31±0.78 | 5.48±0.99 | 6.77±3.18 | <0.01 |
| TC, mmol/L | 5.01±1.38 | 4.93±1.09 | 4.97±1.42 | 5.02±1.56 | 5.11±1.39 | <0.01 |
| †TG, mmol/L | 1.29 (0.92,1.92) | 1.23 (0.87,1.80) | 1.25 (0.90,1.85) | 1.29 (0.92,1.90) | 1.40 (1.00,2.11) | <0.01 |
| LDL-C, mmol/L | 2.62±1.07 | 2.60±0.90 | 2.62±1.08 | 2.61±1.20 | 2.64±1.10 | <0.01 |
| HDL-C, mmol/L | 1.54±0.53 | 1.53±0.51 | 1.54±0.51 | 1.55±0.51 | 1.54±0.58 | <0.01 |
| Scr, μmol/L | 82.56±28.18 | 81.75±23.59 | 81.99±26.73 | 82.44±27.71 | 84.07±33.73 | <0.01 |
| eGFR, mL/min | 88.76±20.83 | 89.96±21.13 | 89.60±20.13 | 88.62±19.89 | 86.87±21.96 | <0.01 |
| The number of FPG measurements |  |  |  |  |  | <0.01 |
| 2 | 41828 (42.61) | 11966 (48.44) | 8556 (35.36) | 9503 (38.26) | 11803 (48.33) |  |
| 3 | 56333 (57.39) | 12738 (51.56) | 15641 (64.64) | 15337 (61.74) | 12617 (51.67) |  |
| FPG Variability |  |  |  |  |  |  |
| †SD | 0.43 (0.24,0.71) | 0.14 (0.08,0.20) | 0.33 (0.27,0.40) | 0.53 (0.45,0.63) | 1.03 (0.80,1.54) | <0.01 |
| †VIM | 0.43 (0.24,0.71) | 0.14 (0.08,0.20) | 0.33 (0.27,0.40) | 0.53 (0.45,0.63) | 1.03 (0.80,1.54) | <0.01 |
| †CV | 8.14 (4.56,13.20) | 2.70 (1.50,3.82) | 6.34 (5.17,7.77) | 10.14(8.48,12.13) | 18.06 (14.33,24.07) | <0.01 |

Note: Q1, the average real variability of FPG < 0.30; Q2, 0.30 ≤ the average real variability of FPG < 0.54; Q3, 0.54 ≤ the average real variability of FPG < 0.94; Q4, the average real variability of FPG ≥ 0.94.

Low-income level: income≥800 Renminbi/month.

Continuous variables are presented as mean±SD, and categorical variables are presented as percentage.

Abbreviations: ARV, average real variability; BMI, body mass index; CKD, chronic kidney disease; CV, coefficient of variance; DBP, diastolic blood pressure; eGFR, estimated glomerular filtration rate; FPG, fasting plasma glucose; HDL-C, high-density lipoprotein cholesterol; LDL-C, low-density lipoprotein cholesterol; RHR, resting heart rate; SBP, systolic blood pressure; SD, standard deviation; Scr, serum creatinine levels; TC, total cholesterol; TG, triglyceride; VIM, variability independent of mean.

* The eGFR was calculated using the Chronic Kidney Disease Epidemiology Collaboration equation. Chronic kidney disease; CKD was defined as an eGFR (calculated using the Modification of Diet in Renal Disease formula) under 60 mL/min at the baseline of health examination.

† Triglyceride levels and the SD, CV, ARV, VIM of fasting plasma glucose levels are presented as median (interquartile range).

**Table S4. Risks of total digestive cancers according to quartiles of FPG variability assessed by CV**

|  | **HR (95% CI) according to quartiles of variability of FPG** | | | |
| --- | --- | --- | --- | --- |
| **Variable** | **Quartile 1** | **Quartile 2** | **Quartile 3** | **Quartile 4** |
| Event, n (%) | 221 (0.90) | 253 (1.03) | 287 (1.17) | 342 (1.39) |
| IR* | 0.96 | 1.12 | 1.26 | 1.49 |
| Cox regression models |  |  |  |  |
| Model 1  P value | 1(reference)  0.0003† | 1.354(1.130-1.622)  0.001 | 1.430(1.200-1.705)  <0.0001 | 1.367(1.154-1.620)  0.0003 |
| Model 2  P value | 1(reference)  0.0004† | 1.354(1.131-1.623)  0.001 | 1.426(1.196-1.700)  <0.0001 | 1.341(1.128-1.594)  0.0009 |
| Model 3  P value | 1(reference)  0.0004† | 1.353(1.129-1.621)  0.001 | 1.425(1.195-1.698)  <0.0001 | 1.338(1.125-1.591)  0.0010 |

*IR (incidence rate) presented as per 1000 person-years

†P for trend

Model 1: adjusted for age and sex

Model 2: Model 1 + LDL-C, baseline FPG, antihypertensive drugs, hypoglycemic drugs, hypertension and diabetes mellitus

Model 3: Model 2 + BMI, current smoking, current drinking, physical exercise and family history of cancer

Abbreviations: CI, confidence interval; CV, coefficient of variance; FPG, fasting plasma glucose; HR, hazard ratio.

**Table S5. Risks of total digestive cancers according to quartiles of FPG variability assessed by VIM**

|  | **HR (95% CI) according to quartiles of variability of FPG** | | | |
| --- | --- | --- | --- | --- |
| **Variable** | **Quartile 1** | **Quartile 2** | **Quartile 3** | **Quartile 4** |
| Event, n (%) | 213 (0.99) | 244 (1.25) | 339 (1.21) | 307 (1.42) |
| IR* | 0.92 | 1.07 | 1.34 | 1.52 |
| Cox regression models |  |  |  |  |
| Model 1  P value | 1(reference)  <0.0001† | 1.195(1.085-1.317)  0.0003 | 1.232(1.123-1.352)  <0.0001 | 1.108(1.007-1.219)  0.0360 |
| Model 2  P value | 1(reference)  <0.0001† | 1.343(1.117-1.614)  0.0017 | 1.561(1.314-1.853)  <0.0001 | 1.362(1.133-1.637)  0.0010 |
| Model 3  P value | 1(reference)  <0.0001† | 1.342(1.116-1.613)  0.0017 | 1.558(1.312-1.85)  <0.0001 | 1.358(1.129-1.633)  0.0011 |

*IR (incidence rate) presented as per 1000 person-years

†P for trend

Model 1: adjusted for age and sex

Model 2: Model 1 + LDL-C, baseline FPG, antihypertensive drugs, hypoglycemic drugs, hypertension and diabetes mellitus

Model 3: Model 2 + BMI, current smoking, current drinking, physical exercise and family history of cancer

Abbreviations: CI, confidence interval; FPG, fasting plasma glucose; HR, hazard ratio; VIM, variability independent of mean.

**Table S6. Risks of total digestive cancers according to quartiles of FPG variability assessed by ARV**

|  | **HR (95% CI) according to quartiles of variability of FPG** | | | |
| --- | --- | --- | --- | --- |
| **Variable** | **Quartile 1** | **Quartile 2** | **Quartile 3** | **Quartile 4** |
| Event, n (%) | 223(0.90) | 230(0.95) | 309(1.24) | 341(1.40) |
| IR* | 0.97 | 1.03 | 1.34 | 1.48 |
| Cox regression models |  |  |  |  |
| Model 1  P value | 1(reference)  0.0050† | 1.181(0.982-1.419)  0.0776 | 1.369(1.153-1.627)  0.0004 | 1.185(1.000-1.404)  0.0496 |
| Model 2  P value | 1(reference)  0.0048† | 1.180(0.981-1.419)  0.0785 | 1.364(1.148-1.621)  0.0004 | 1.142(0.957-1.362)  0.1405 |
| Model 3  P value | 1(reference)  0.0051† | 1.177(0.979-1.416)  0.0826 | 1.361(1.145-1.618)  0.0005 | 1.138(0.954-1.358)  0.1509 |

*IR (incidence rate) presented as per 1000 person-years

†P for trend

Model 1: adjusted for age and sex

Model 2: Model 1 + LDL-C, baseline FPG, antihypertensive drugs, hypoglycemic drugs, hypertension and diabetes mellitus

Model 3: Model 2 + BMI, current smoking, current drinking, physical exercise and family history of cancer

Abbreviations: ARV, average real variability; CI, confidence interval; FPG, fasting plasma glucose; HR, hazard ratio.

**Table S7. Risks of site-specific digestive cancer according to quartiles of FPG variability assessed by CV**

|  | **HR (95% CI) for Quartiles of Variability of Fasting Plasma Glucose** | | | |
| --- | --- | --- | --- | --- |
| **Variables** | **Quartile 1** | **Quartile 2** | **Quartile 3** | **Quartile 4** |
| **Gastric and Esophageal** |  |  |  |  |
| Events, n (%) | 54(0.22) | 46(0.19) | 54(0.22) | 71(0.29) |
| IR* | 0.22 | 0.19 | 0.22 | 0.29 |
| Cox regression models |  |  |  |  |
| Model 1  P value | 1(reference)  0.8656† | 1.014(0.684-1.504)  0.9432 | 1.104(0.756-1.610)  0.6094 | 1.143(0.801-1.630)  0.4608 |
| Model 2  P value | 1(reference)  0.9005† | 1.014(0.684-1.503)  0.9453 | 1.104(0.756-1.611)  0.6093 | 1.125(0.783-1.617)  0.5234 |
| Model 3  P value | 1(reference)  0.9146† | 1.009(0.68-1.495)  0.9654 | 1.098(0.752-1.602)  0.6295 | 1.115(0.776-1.603)  0.5565 |
| **Liver** |  |  |  |  |
| Events, n (%) | 44(0.18) | 61(0.25) | 73(0.3) | 65(0.26) |
| IR* | 0.18 | 0.25 | 0.3 | 0.26 |
| Cox regression models |  |  |  |  |
| Model 1  P value | 1(reference)  0.0075† | 1.654(1.122-2.438)  0.0111 | 1.874(1.289-2.726)  0.0010 | 1.364(0.929-2.002)  0.1127 |
| Model 2  P value | 1(reference)  0.0071† | 1.653(1.121-2.437)  0.0111 | 1.862(1.28-2.709)  0.0012 | 1.324(0.897-1.955)  0.1582 |
| Model 3  P value | 1(reference)  0.0069† | 1.65(1.119-2.433)  0.0114 | 1.861(1.279-2.707)  0.0012 | 1.315(0.89-1.942)  0.1694 |
| **Pancreatic** |  |  |  |  |
| Events, n (%) | 12(0.05) | 25(0.1) | 18(0.07) | 28(0.11) |
| IR* | 0.05 | 0.10 | 0.07 | 0.11 |
| Cox regression models |  |  |  |  |
| Model 1  P value | 1(reference)  0.0756† | 2.437(1.224-4.853)  0.0113 | 1.61(0.775-3.346)  0.2017 | 1.977(1.004-3.893)  0.0488 |
| Model 2  P value | 1(reference)  0.0798† | 2.438(1.224-4.857)  0.0112 | 1.601(0.77-3.328)  0.2075 | 1.901(0.955-3.786)  0.0675 |
| Model 3  P value | 1(reference)  0.0806† | 2.435(1.223-4.851)  0.0114 | 1.608(0.773-3.342)  0.2034 | 1.914(0.961-3.811)  0.0648 |
| **Colorectal** |  |  |  |  |
| Events, n (%) | 84(0.34) | 90(0.37) | 110(0.45) | 135(0.55) |
| IR* | 0.34 | 0.37 | 0.45 | 0.55 |
| Cox regression models |  |  |  |  |
| Model 1  P value | 1(reference)  0.0549† | 1.259(0.935-1.696)  0.1292 | 1.428(1.074-1.898)  0.0141 | 1.407(1.07-1.849)  0.0144 |
| Model 2  P value | 1(reference)  0.0557† | 1.259(0.935-1.695)  0.1298 | 1.433(1.078-1.905)  0.0133 | 1.409(1.066-1.861)  0.0160 |
| Model 3  P value | 1(reference)  0.0622† | 1.257(0.933-1.692)  0.1327 | 1.426(1.073-1.896)  0.0146 | 1.400(1.059-1.851)  0.0180 |

*IR (incidence rate) presented as per 1000 person-years

†P for trend

Model 1: adjusted for age and sex

Model 2: Model 1 + LDL-C, baseline FPG, antihypertensive drugs, hypoglycemic drugs, hypertension and diabetes mellitus

Model 3: Model 2 + BMI, current smoking, current drinking, physical exercise and family history of cancer

Abbreviations: CI, confidence interval; CV, coefficient of variance; FPG, fasting plasma glucose; HR, hazard ratio.

**Table S8. Risks of site-specific digestive cancer according to quartiles of FPG variability assessed by VIM**

|  | **HR (95% CI) according to quartiles of variability of FPG** | | | |
| --- | --- | --- | --- | --- |
| **Variables** | **Quartile 1** | **Quartile 2** | **Quartile 3** | **Quartile 4** |
| **Gastric and Esophageal** |  |  |  |  |
| Events, n (%) | 50(0.2) | 47(0.19) | 66(0.24) | 62(0.29) |
| IR* | 0.20 | 0.19 | 0.24 | 0.29 |
| Cox regression models |  |  |  |  |
| Model 1  P value | 1(reference)  0.5612† | 1.109(0.745-1.653)  0.6102 | 1.299(0.899-1.877)  0.1643 | 1.186(0.816-1.724)  0.3713 |
| Model 2  P value | 1(reference)  0.5740† | 1.108(0.744-1.65)  0.6149 | 1.298(0.898-1.876)  0.1657 | 1.145(0.772-1.697)  0.5014 |
| Model 3  P value | 1(reference)  0.5991† | 1.100(0.738-1.640)  0.6384 | 1.287(0.890-1.860)  0.1802 | 1.134(0.764-1.682)  0.5325 |
| **Liver** |  |  |  |  |
| Events, n (%) | 46(0.19) | 55(0.22) | 76(0.28) | 66(0.31) |
| IR* | 0.19 | 0.22 | 0.28 | 0.31 |
| Cox regression models |  |  |  |  |
| Model 1  P value | 1(reference)  0.0522† | 1.413(0.955-2.092)  0.0839 | 1.674(1.16-2.416)  0.0059 | 1.461(1.001-2.132)  0.0492 |
| Model 2  P value | 1(reference)  0.0588† | 1.412(0.954-2.09)  0.0848 | 1.664(1.153-2.402)  0.0066 | 1.43(0.966-2.117)  0.0740 |
| Model 3  P value | 1(reference)  0.0580† | 1.408(0.951-2.084)  0.0875 | 1.668(1.155-2.409)  0.0063 | 1.411(0.952-2.092)  0.0860 |
| **Pancreatic** |  |  |  |  |
| Events, n (%) | 11(0.04) | 26(0.11) | 21(0.08) | 25(0.12) |
| IR* | 0.04 | 0.11 | 0.08 | 0.12 |
| Cox regression models |  |  |  |  |
| Model 1  P value | 1(reference)  0.0436† | 2.743(1.355-5.555)  0.0051 | 1.825(0.879-3.789)  0.1065 | 2.114(1.038-4.302)  0.0390 |
| Model 2  P value | 1(reference)  0.0454† | 2.743(1.354-5.556)  0.0051 | 1.814(0.873-3.767)  0.1105 | 1.967(0.939-4.123)  0.0731 |
| Model 3  P value | 1(reference)  0.0456† | 2.745(1.355-5.56)  0.0050 | 1.827(0.879-3.797)  0.1061 | 1.983(0.946-4.158)  0.0700 |
| **Colorectal** |  |  |  |  |
| Events, n (%) | 82(0.33) | 82(0.33) | 136(0.5) | 119(0.55) |
| IR* | 0.33 | 0.33 | 0.5 | 0.55 |
| Cox regression models |  |  |  |  |
| Model 1  P value | 1(reference)  0.0038† | 1.166(0.858-1.584)  0.3267 | 1.611(1.224-2.12)  0.0007 | 1.406(1.061-1.865)  0.0179 |
| Model 2  P value | 1(reference)  0.0037† | 1.166(0.858-1.584)  0.3253 | 1.619(1.23-2.131)  0.0006 | 1.411(1.047-1.9)  0.0236 |
| Model 3  P value | 1(reference)  0.0046† | 1.164(0.857-1.582)  0.3311 | 1.607(1.221-2.115)  0.0007 | 1.4(1.039-1.887)  0.0272 |

*IR (incidence rate) presented as per 1000 person-years

†P for trend

Model 1: adjusted for age and sex

Model 2: Model 1 + LDL-C, baseline FPG, antihypertensive drugs, hypoglycemic drugs, hypertension and diabetes mellitus

Model 3: Model 2 + BMI, current smoking, current drinking, physical exercise and family history of cancer

Abbreviations: CI, confidence interval; FPG, fasting plasma glucose; HR, hazard ratio; VIM, variability independent of mean.

**Table S9. Risks of site-specific digestive cancer according to quartiles of FPG variability assessed by ARV**

|  | **HR (95% CI) according to quartiles of variability of FPG** | | | |
| --- | --- | --- | --- | --- |
| **Variables** | **Quartile 1** | **Quartile 2** | **Quartile 3** | **Quartile 4** |
| **Gastric and Esophageal** |  |  |  |  |
| Events, n (%) | 54(0.22) | 41(0.17) | 58(0.23) | 72(0.29) |
| IR* | 0.22 | 0.17 | 0.23 | 0.29 |
| Cox regression models |  |  |  |  |
| Model 1  P value | 1(reference)  0.8160† | 0.872(0.581-1.309)  0.5086 | 1.058(0.730-1.533)  0.7671 | 1.007(0.707-1.436)  0.9671 |
| Model 2  P value | 1(reference)  0.8156† | 0.870(0.579-1.306)  0.5006 | 1.057(0.729-1.532)  0.7699 | 0.973(0.672-1.409)  0.8860 |
| Model 3  P value | 1(reference)  0.8021† | 0.859(0.572-1.290)  0.4642 | 1.047(0.722-1.519)  0.8070 | 0.963(0.665-1.394)  0.8402 |
| **Liver** |  |  |  |  |
| Events, n (%) | 48(0.19) | 52(0.21) | 69(0.28) | 74(0.3) |
| IR* | 0.19 | 0.21 | 0.28 | 0.30 |
| Cox regression models |  |  |  |  |
| Model 1  P value | 1(reference)  0.2713† | 1.247(0.842-1.846)  0.2710 | 1.451(1.003-2.097)  0.04800 | 1.242(0.863-1.789)  0.2435 |
| Model 2  P value | 1(reference)  0.2861† | 1.246(0.841-1.845)  0.2726 | 1.440(0.995-2.082)  0.0529 | 1.212(0.832-1.766)  0.3157 |
| Model 3  P value | 1(reference)  0.2875† | 1.242(0.839-1.840)  0.2789 | 1.438(0.994-2.080)  0.0538 | 1.204(0.826-1.755)  0.3353 |
| **Pancreatic** |  |  |  |  |
| Events, n (%) | 13(0.05) | 20(0.08) | 24(0.1) | 26(0.11) |
| IR* | 0.05 | 0.08 | 0.10 | 0.11 |
| Cox regression models |  |  |  |  |
| Model 1  P value | 1(reference)  0.3587† | 1.738(0.864-3.496)  0.1210 | 1.774(0.903-3.486)  0.0963 | 1.482(0.760-2.889)  0.2483 |
| Model 2  P value | 1(reference)  0.3407† | 1.730(0.860-3.481)  0.1242 | 1.759(0.894-3.458)  0.1017 | 1.363(0.683-2.722)  0.3800 |
| Model 3  P value | 1(reference)  0.3376† | 1.733(0.861-3.487)  0.1233 | 1.767(0.899-3.476)  0.0988 | 1.372(0.687-2.743)  0.3701 |
| **Colorectal** |  |  |  |  |
| Events, n (%) | 87(0.35) | 77(0.32) | 123(0.5) | 132(0.54) |
| IR* | 0.35 | 0.32 | 0.5 | 0.54 |
| Cox regression models |  |  |  |  |
| Model 1  P value | 1(reference)  0.0568† | 1.012(0.745-1.376)  0.9382 | 1.394(1.059-1.836)  0.0177 | 1.180(0.899-1.549)  0.2322 |
| Model 2  P value | 1(reference)  0.0538† | 1.011(0.744-1.375)  0.9423 | 1.399(1.063-1.842)  0.0167 | 1.167(0.878-1.550)  0.2880 |
| Model 3  P value | 1(reference)  0.0600† | 1.008(0.742-1.371)  0.9570 | 1.390(1.056-1.830)  0.0190 | 1.157(0.871-1.538)  0.3146 |

*IR (incidence rate) presented as per 1000 person-years

†P for trend

Model 1: adjusted for age and sex

Model 2: Model 1 + LDL-C, baseline FPG, antihypertensive drugs, hypoglycemic drugs, hypertension and diabetes mellitus

Model 3: Model 2 + BMI, current smoking, current drinking, physical exercise and family history of cancer

Abbreviations: ARV, average real variability; CI, confidence interval; FPG, fasting plasma glucose; HR, hazard ratio.

**Table S10. Subgroup analyses according to quartiles of FPG variability assessed by SD**

| **Variables** | **Group** | **Model 1**  **HR (95%CI)** | **P value** | **Model 2**  **HR (95%CI)** | **P value** | **Model 3**  **HR (95%CI)** | **P value** |
| --- | --- | --- | --- | --- | --- | --- | --- |
| **Age** |  |  | 0.0034† |  | 0.0032† |  | 0.0031† |
| <65 y | Q1 | 1(reference) |  | 1(reference) |  | 1(reference) |  |
|  | Q2 | 1.396(1.117-1.746) | 0.0034 | 1.396(1.117-1.746) | 0.0034 | 1.393(1.114-1.742) | 0.0037 |
|  | Q3 | 1.596(1.286-1.982) | <.0001 | 1.593(1.283-1.979) | <.0001 | 1.591(1.281-1.976) | <.0001 |
|  | Q4 | 1.663(1.354-2.044) | <.0001 | 1.615(1.302-2.002) | <.0001 | 1.616(1.303-2.004) | <.0001 |
| ≥65 y | Q1 | 1(reference) |  | 1(reference) |  | 1(reference) |  |
|  | Q2 | 1.188(0.859-1.643) | 0.2965 | 1.194(0.863-1.651) | 0.2849 | 1.19(0.860-1.646) | 0.2937 |
|  | Q3 | 1.374(1.015-1.86) | 0.0399 | 1.373(1.013-1.86) | 0.0408 | 1.365(1.007-1.849) | 0.0450 |
|  | Q4 | 0.9(0.661-1.225) | 0.5027 | 0.917(0.664-1.266) | 0.5967 | 0.918(0.664-1.269) | 0.6035 |
| **Sex** |  |  | 0.1644† |  | 0.1607† |  | 0.1604† |
| Male | Q1 | 1(reference) |  | 1(reference) |  | 1(reference) |  |
|  | Q2 | 1.429(1.177-1.736) | 0.0003 | 1.43(1.177-1.737) | 0.0003 | 1.429(1.176-1.736) | 0.0003 |
|  | Q3 | 1.569(1.3-1.894) | <.0001 | 1.567(1.298-1.891) | <.0001 | 1.565(1.296-1.889) | <.0001 |
|  | Q4 | 1.471(1.227-1.765) | <.0001 | 1.425(1.179-1.721) | 0.0002 | 1.422(1.177-1.719) | 0.0003 |
| Female | Q1 | 1(reference) |  | 1(reference) |  | 1(reference) |  |
|  | Q2 | 0.775(0.428-1.403) | 0.4000 | 0.778(0.429-1.41) | 0.408 | 0.784(0.432-1.421) | 0.4223 |
|  | Q3 | 1.469(0.892-2.419) | 0.1312 | 1.487(0.901-2.454) | 0.1206 | 1.492(0.904-2.463) | 0.1179 |
|  | Q4 | 1.115(0.665-1.867) | 0.6803 | 1.247(0.722-2.152) | 0.4288 | 1.257(0.727-2.172) | 0.4127 |
| **Diabetes mellitus** |  |  | 0.1928† |  | 0.1980† |  | 0.1964† |
| Yes | Q1 | 1(reference) |  | 1(reference) |  | 1(reference) |  |
|  | Q2 | 1.491(0.473-4.7) | 0.4949 | 1.429(0.453-4.513) | 0.5425 | 1.374(0.433-4.359) | 0.5891 |
|  | Q3 | 0.497(0.119-2.079) | 0.338 | 0.459(0.11-1.926) | 0.2873 | 0.438(0.104-1.84) | 0.2595 |
|  | Q4 | 1.448(0.576-3.642) | 0.4314 | 1.293(0.508-3.292) | 0.5893 | 1.287(0.505-3.28) | 0.5967 |
| No | Q1 | 1(reference) |  | 1(reference) |  | 1(reference) |  |
|  | Q2 | 1.338(1.111-1.613) | 0.0022 | 1.339(1.111-1.613) | 0.0022 | 1.337(1.11-1.611) | 0.0023 |
|  | Q3 | 1.589(1.331-1.898) | <.0001 | 1.589(1.33-1.898) | <.0001 | 1.584(1.326-1.892) | <.0001 |
|  | Q4 | 1.383(1.158-1.653) | 0.0003 | 1.364(1.134-1.64) | 0.0010 | 1.358(1.13-1.634) | 0.0011 |
| **BMI** |  |  | 0.3856† |  | 0.3762† |  | 0.3768† |
| <25 kg/m^2^ | Q1 | 1(reference) |  | 1(reference) |  | 1(reference) |  |
|  | Q2 | 1.306(1.017-1.675) | 0.0361 | 1.299(1.012-1.666) | 0.0401 | 1.295(1.009-1.662) | 0.0422 |
|  | Q3 | 1.491(1.171-1.899) | 0.0012 | 1.478(1.16-1.883) | 0.0016 | 1.471(1.155-1.874) | 0.0018 |
|  | Q4 | 1.551(1.231-1.956) | 0.0002 | 1.505(1.182-1.915) | 0.0009 | 1.495(1.174-1.903) | 0.0011 |
| ≥25 kg/m^2^ | Q1 | 1(reference) |  | 1(reference) |  | 1(reference) |  |
|  | Q2 | 1.39(1.058-1.825) | 0.0181 | 1.388(1.056-1.824) | 0.0186 | 1.388(1.057-1.824) | 0.0185 |
|  | Q3 | 1.629(1.258-2.108) | 0.0002 | 1.625(1.256-2.104) | 0.0002 | 1.627(1.257-2.106) | 0.0002 |
|  | Q4 | 1.301(1.008-1.678) | 0.043 | 1.272(0.976-1.659) | 0.0755 | 1.274(0.977-1.663) | 0.0742 |

*IR (incidence rate) presented as per 1000 person-years

†P for interaction

Model 1: adjusted for age and sex

Model 2: Model 1 + LDL-C, baseline FPG, antihypertensive drugs, hypoglycemic drugs, hypertension and diabetes mellitus

Model 3: Model 2 + BMI, current smoking, current drinking, physical exercise and family history of cancer

Abbreviations: BMI, body mass index; CI, confidence interval; FPG, fasting plasma glucose; HR, hazard ratio; SD, standard deviation.

**Table S11. Subgroup analyses according to quartiles of FPG variability assessed by CV**

| **Variables** | **Group** | **Model 1**  **HR (95%CI)** | **P value** | **Model 2**  **HR (95%CI)** | **P value** | **Model 3**  **HR (95%CI)** | **P value** |
| --- | --- | --- | --- | --- | --- | --- | --- |
| **Age** |  |  | 0.0063† |  | 0.0066† |  | 0.0061† |
| <65 y | Q1 | 1(reference) |  | 1(reference) |  | 1(reference) |  |
|  | Q2 | 1.513(1.216-1.882) | 0.0002 | 1.515(1.217-1.885) | 0.0002 | 1.511(1.215-1.88) | 0.0002 |
|  | Q3 | 1.489(1.198-1.85) | 0.0003 | 1.48(1.191-1.84) | 0.0004 | 1.479(1.19-1.838) | 0.0004 |
|  | Q4 | 1.616(1.315-1.987) | <.0001 | 1.569(1.271-1.937) | <.0001 | 1.567(1.27-1.935) | <.0001 |
| ≥65 y | Q1 | 1(reference) |  | 1(reference) |  | 1(reference) |  |
|  | Q2 | 1.027(0.743-1.42) | 0.8714 | 1.033(0.747-1.428) | 0.8451 | 1.033(0.747-1.428) | 0.8455 |
|  | Q3 | 1.235(0.916-1.665) | 0.1665 | 1.233(0.914-1.663) | 0.1706 | 1.234(0.914-1.665) | 0.1694 |
|  | Q4 | 0.88(0.653-1.185) | 0.4000 | 0.902(0.666-1.223) | 0.5084 | 0.905(0.667-1.227) | 0.5192 |
| **Sex** |  |  | 0.2013† |  | 0.1970† |  | 0.1979† |
| Male | Q1 | 1(reference) |  | 1(reference) |  | 1(reference) |  |
|  | Q2 | 1.436(1.186-1.737) | 0.0002 | 1.437(1.188-1.739) | 0.0002 | 1.436(1.186-1.738) | 0.0002 |
|  | Q3 | 1.44(1.194-1.737) | 0.0001 | 1.437(1.191-1.733) | 0.0002 | 1.436(1.19-1.732) | 0.0002 |
|  | Q4 | 1.41(1.177-1.688) | 0.0002 | 1.376(1.146-1.654) | 0.0006 | 1.374(1.144-1.651) | 0.0007 |
| Female | Q1 | 1(reference) |  | 1(reference) |  | 1(reference) |  |
|  | Q2 | 0.812(0.453-1.456) | 0.4848 | 0.821(0.457-1.475) | 0.5094 | 0.829(0.461-1.489) | 0.5297 |
|  | Q3 | 1.361(0.821-2.257) | 0.2314 | 1.387(0.835-2.303) | 0.2061 | 1.395(0.84-2.319) | 0.1985 |
|  | Q4 | 1.087(0.653-1.812) | 0.7481 | 1.14(0.675-1.927) | 0.6234 | 1.155(0.683-1.953) | 0.5912 |
| **Diabetes mellitus** |  |  | 0.1967† |  | 0.1924† |  | 0.1824† |
| Yes | Q1 | 1(reference) |  | 1(reference) |  | 1(reference) |  |
|  | Q2 | 0.443(0.139-1.414) | 0.1691 | 0.441(0.138-1.407) | 0.1664 | 0.426(0.133-1.361) | 0.1498 |
|  | Q3 | 0.859(0.364-2.026) | 0.7284 | 0.824(0.349-1.947) | 0.6587 | 0.815(0.344-1.93) | 0.6425 |
|  | Q4 | 1.076(0.537-2.159) | 0.8357 | 1.012(0.504-2.035) | 0.9722 | 1.03(0.512-2.072) | 0.9331 |
| No | Q1 | 1(reference) |  | 1(reference) |  | 1(reference) |  |
|  | Q2 | 1.399(1.165-1.681) | 0.0003 | 1.401(1.166-1.683) | 0.0003 | 1.399(1.164-1.681) | 0.0003 |
|  | Q3 | 1.454(1.215-1.74) | <.0001 | 1.454(1.215-1.74) | <.0001 | 1.451(1.212-1.736) | <.0001 |
|  | Q4 | 1.347(1.129-1.606) | 0.001 | 1.331(1.112-1.593) | 0.0018 | 1.326(1.108-1.587) | 0.0021 |
| **BMI** |  |  | 0.6529† |  | 0.6434† |  | 0.6424† |
| <25 kg/m^2^ | Q1 | 1(reference) |  | 1(reference) |  | 1(reference) |  |
|  | Q2 | 1.485(1.16-1.9) | 0.0017 | 1.478(1.155-1.892) | 0.0019 | 1.474(1.151-1.886) | 0.0021 |
|  | Q3 | 1.477(1.156-1.887) | 0.0018 | 1.459(1.141-1.865) | 0.0026 | 1.453(1.137-1.858) | 0.0028 |
|  | Q4 | 1.505(1.189-1.904) | 0.0007 | 1.456(1.146-1.849) | 0.0021 | 1.446(1.139-1.837) | 0.0025 |
| ≥25 kg/m^2^ | Q1 | 1(reference) |  | 1(reference) |  | 1(reference) |  |
|  | Q2 | 1.224(0.938-1.596) | 0.1369 | 1.221(0.936-1.593) | 0.1418 | 1.221(0.936-1.593) | 0.1411 |
|  | Q3 | 1.377(1.07-1.772) | 0.0128 | 1.377(1.07-1.772) | 0.0129 | 1.38(1.072-1.776) | 0.0123 |
|  | Q4 | 1.228(0.961-1.569) | 0.1013 | 1.218(0.948-1.564) | 0.1236 | 1.22(0.949-1.567) | 0.1205 |

*IR (incidence rate) presented as per 1000 person-years

†P for interaction

Model 1: adjusted for age and sex

Model 2: Model 1 + LDL-C, baseline FPG, antihypertensive drugs, hypoglycemic drugs, hypertension and diabetes mellitus

Model 3: Model 2 + BMI, current smoking, current drinking, physical exercise and family history of cancer

Abbreviations: BMI, body mass index; CI, confidence interval; CV, coefficient of variance; FPG, fasting plasma glucose; HR, hazard ratio.

**Table S12. Subgroup analyses according to quartiles of FPG variability assessed by VIM**

| **Variables** | **Group** | **Model 1**  **HR (95%CI)** | **P value** | **Model 2**  **HR (95%CI)** | **P value** | **Model 3**  **HR (95%CI)** | **P value** |
| --- | --- | --- | --- | --- | --- | --- | --- |
| **Age** |  |  | 0.0009† |  | 0.0009† |  | 0.0008† |
| <65 y | Q1 | 1(reference) |  | 1(reference) |  | 1(reference) |  |
|  | Q2 | 1.397(1.117-1.747) | 0.0034 | 1.397(1.117-1.747) | 0.0034 | 1.393(1.114-1.743) | 0.0036 |
|  | Q3 | 1.598(1.294-1.973) | <.0001 | 1.594(1.29-1.969) | <.0001 | 1.59(1.287-1.964) | <.0001 |
|  | Q4 | 1.671(1.354-2.061) | <.0001 | 1.618(1.298-2.018) | <.0001 | 1.622(1.301-2.023) | <.0001 |
| ≥65 y | Q1 | 1(reference) |  | 1(reference) |  | 1(reference) |  |
|  | Q2 | 1.189(0.86-1.645) | 0.294 | 1.194(0.864-1.652) | 0.2830 | 1.19(0.861-1.647) | 0.2923 |
|  | Q3 | 1.389(1.034-1.867) | 0.029 | 1.387(1.031-1.865) | 0.0304 | 1.378(1.025-1.854) | 0.0339 |
|  | Q4 | 0.837(0.608-1.154) | 0.2775 | 0.844(0.603-1.181) | 0.3231 | 0.845(0.603-1.185) | 0.3291 |
| **Sex** |  |  | 0.1950† |  | 0.1909† |  | 0.1907† |
| Male | Q1 | 1(reference) |  | 1(reference) |  | 1(reference) |  |
|  | Q2 | 1.43(1.177-1.737) | 0.0003 | 1.431(1.178-1.738) | 0.0003 | 1.43(1.177-1.737) | 0.0003 |
|  | Q3 | 1.586(1.32-1.905) | <.0001 | 1.582(1.317-1.901) | <.0001 | 1.58(1.315-1.898) | <.0001 |
|  | Q4 | 1.445(1.199-1.741) | 0.0001 | 1.389(1.143-1.688) | 0.0010 | 1.387(1.141-1.686) | 0.0010 |
| Female | Q1 | 1(reference) |  | 1(reference) |  | 1(reference) |  |
|  | Q2 | 0.775(0.428-1.404) | 0.4002 | 0.778(0.429-1.41) | 0.4078 | 0.784(0.432-1.421) | 0.4222 |
|  | Q3 | 1.421(0.87-2.321) | 0.1606 | 1.44(0.88-2.357) | 0.1463 | 1.449(0.885-2.372) | 0.1402 |
|  | Q4 | 1.126(0.664-1.91) | 0.6607 | 1.28(0.728-2.251) | 0.3905 | 1.287(0.731-2.263) | 0.3820 |
| **Diabetes mellitus** |  |  | 0.2334† |  | 0.2414† |  | 0.2401† |
| Yes | Q1 | 1(reference) |  | 1(reference) |  | 1(reference) |  |
|  | Q2 | 1.521(0.483-4.794) | 0.4739 | 1.461(0.462-4.613) | 0.5185 | 1.412(0.445-4.483) | 0.5581 |
|  | Q3 | 0.688(0.199-2.377) | 0.5541 | 0.642(0.185-2.222) | 0.4843 | 0.613(0.177-2.125) | 0.4400 |
|  | Q4 | 1.46(0.58-3.678) | 0.422 | 1.299(0.509-3.311) | 0.5840 | 1.299(0.509-3.316) | 0.5839 |
| No | Q1 | 1(reference) |  | 1(reference) |  | 1(reference) |  |
|  | Q2 | 1.339(1.111-1.613) | 0.0022 | 1.339(1.111-1.614) | 0.0021 | 1.338(1.11-1.612) | 0.0022 |
|  | Q3 | 1.592(1.339-1.894) | <.0001 | 1.591(1.337-1.892) | <.0001 | 1.586(1.333-1.886) | <.0001 |
|  | Q4 | 1.355(1.128-1.627) | 0.0012 | 1.329(1.098-1.608) | 0.0034 | 1.324(1.094-1.602) | 0.0040 |
| **BMI** |  |  | 0.6791† |  | 0.6644† |  | 0.6634† |
| <25 kg/m^2^ | Q1 | 1(reference) |  | 1(reference) |  | 1(reference) |  |
|  | Q2 | 1.307(1.018-1.677) | 0.0354 | 1.3(1.013-1.668) | 0.0393 | 1.296(1.01-1.664) | 0.0415 |
|  | Q3 | 1.543(1.221-1.949) | 0.0003 | 1.527(1.208-1.93) | 0.0004 | 1.519(1.202-1.921) | 0.0005 |
|  | Q4 | 1.505(1.185-1.911) | 0.0008 | 1.447(1.127-1.858) | 0.0038 | 1.438(1.119-1.846) | 0.0045 |
| ≥25 kg/m^2^ | Q1 | 1(reference) |  | 1(reference) |  | 1(reference) |  |
|  | Q2 | 1.39(1.058-1.826) | 0.0181 | 1.388(1.057-1.824) | 0.0185 | 1.389(1.057-1.825) | 0.0185 |
|  | Q3 | 1.59(1.234-2.05) | 0.0003 | 1.588(1.232-2.047) | 0.0004 | 1.59(1.233-2.049) | 0.0003 |
|  | Q4 | 1.303(1.005-1.689) | 0.0456 | 1.272(0.969-1.669) | 0.0832 | 1.274(0.97-1.672) | 0.0820 |

*IR (incidence rate) presented as per 1000 person-years

†P for interaction

Model 1: adjusted for age and sex

Model 2: Model 1 + LDL-C, baseline FPG, antihypertensive drugs, hypoglycemic drugs, hypertension and diabetes mellitus

Model 3: Model 2 + BMI, current smoking, current drinking, physical exercise and family history of cancer

Abbreviations: BMI, body mass index; CI, confidence interval; FPG, fasting plasma glucose; HR, hazard ratio; VIM, variability independent of mean.

**Table S13. Subgroup analyses according to quartiles of FPG variability assessed by ARV**

| **Variables** | **Group** | **Model 1**  **HR (95%CI)** | **P value** | **Model 2**  **HR (95%CI)** | **P value** | **Model 3**  **HR (95%CI)** | **P value** |
| --- | --- | --- | --- | --- | --- | --- | --- |
| **Age** |  |  | 0.0008† |  | 0.0008† |  | 0.0008† |
| <65 y | Q1 | 1(reference) |  | 1(reference) |  | 1(reference) |  |
|  | Q2 | 1.283(1.028-1.602) | 0.0278 | 1.282(1.027-1.601) | 0.0284 | 1.277(1.023-1.595) | 0.0310 |
|  | Q3 | 1.365(1.102-1.690) | 0.0043 | 1.359(1.098-1.683) | 0.0049 | 1.358(1.097-1.682) | 0.0050 |
|  | Q4 | 1.402(1.142-1.720) | 0.0012 | 1.342(1.084-1.661) | 0.0069 | 1.343(1.085-1.663) | 0.0067 |
| ≥65 y | Q1 | 1(reference) |  | 1(reference) |  | 1(reference) |  |
|  | Q2 | 0.940(0.674-1.311) | 0.7149 | 0.943(0.676-1.315) | 0.7274 | 0.938(0.672-1.309) | 0.7073 |
|  | Q3 | 1.247(0.931-1.671) | 0.1383 | 1.245(0.929-1.669) | 0.1432 | 1.241(0.925-1.664) | 0.1493 |
|  | Q4 | 0.738(0.545-1.000) | 0.0500 | 0.745(0.543-1.021) | 0.0669 | 0.743(0.542-1.02) | 0.0660 |
| **Sex** |  |  | 0.1995† |  | 0.1961† |  | 0.1969† |
| Male | Q1 | 1(reference) |  | 1(reference) |  | 1(reference) |  |
|  | Q2 | 1.257(1.035-1.528) | 0.0213 | 1.256(1.034-1.527) | 0.0217 | 1.254(1.032-1.524) | 0.0230 |
|  | Q3 | 1.387(1.153-1.668) | 0.0005 | 1.382(1.149-1.662) | 0.0006 | 1.38(1.147-1.659) | 0.0006 |
|  | Q4 | 1.226(1.024-1.468) | 0.0266 | 1.173(0.973-1.414) | 0.0939 | 1.171(0.971-1.411) | 0.0984 |
| Female | Q1 | 1(reference) |  | 1(reference) |  | 1(reference) |  |
|  | Q2 | 0.682(0.376-1.238) | 0.2088 | 0.686(0.378-1.245) | 0.2148 | 0.683(0.376-1.241) | 0.2105 |
|  | Q3 | 1.264(0.775-2.06) | 0.3480 | 1.274(0.78-2.08) | 0.3336 | 1.285(0.786-2.101) | 0.3169 |
|  | Q4 | 0.915(0.55-1.524) | 0.7340 | 0.995(0.581-1.703) | 0.9850 | 1(0.584-1.713) | 0.9987 |
| **Diabetes mellitus** |  |  | 0.9037† |  | 0.9201† |  | 0.9154† |
| Yes | Q1 | 1(reference) |  | 1(reference) |  | 1(reference) |  |
|  | Q2 | 1.167(0.356-3.825) | 0.7991 | 1.161(0.353-3.826) | 0.8058 | 1.132(0.342-3.743) | 0.8390 |
|  | Q3 | 1.174(0.383-3.593) | 0.7793 | 1.089(0.355-3.339) | 0.8814 | 1.04(0.338-3.199) | 0.9452 |
|  | Q4 | 1.215(0.481-3.067) | 0.6802 | 1.084(0.424-2.771) | 0.8658 | 1.082(0.422-2.77) | 0.8703 |
| No | Q1 | 1(reference) |  | 1(reference) |  | 1(reference) |  |
|  | Q2 | 1.18(0.979-1.423) | 0.0815 | 1.181(0.98-1.423) | 0.0814 | 1.177(0.977-1.419) | 0.0863 |
|  | Q3 | 1.372(1.153-1.634) | 0.0004 | 1.37(1.15-1.631) | 0.0004 | 1.365(1.146-1.626) | 0.0005 |
|  | Q4 | 1.153(0.967-1.375) | 0.1118 | 1.127(0.94-1.351) | 0.1981 | 1.122(0.935-1.345) | 0.2159 |
| **BMI** |  |  | 0.3764† |  | 0.3619† |  | 0.3609† |
| <25 kg/m^2^ | Q1 | 1(reference) |  | 1(reference) |  | 1(reference) |  |
|  | Q2 | 1.182(0.921-1.517) | 0.1887 | 1.175(0.915-1.508) | 0.2052 | 1.169(0.911-1.501) | 0.2197 |
|  | Q3 | 1.291(1.018-1.637) | 0.0355 | 1.275(1.005-1.618) | 0.0455 | 1.27(1.001-1.611) | 0.0495 |
|  | Q4 | 1.283(1.02-1.614) | 0.0335 | 1.229(0.968-1.559) | 0.0906 | 1.223(0.963-1.552) | 0.0985 |
| ≥25 kg/m^2^ | Q1 | 1(reference) |  | 1(reference) |  | 1(reference) |  |
|  | Q2 | 1.181(0.898-1.553) | 0.2331 | 1.178(0.896-1.55) | 0.2399 | 1.176(0.894-1.546) | 0.2470 |
|  | Q3 | 1.453(1.13-1.869) | 0.0036 | 1.449(1.126-1.863) | 0.0039 | 1.448(1.126-1.863) | 0.0039 |
|  | Q4 | 1.086(0.844-1.397) | 0.5229 | 1.049(0.807-1.362) | 0.7221 | 1.046(0.805-1.359) | 0.7381 |

*IR (incidence rate) presented as per 1000 person-years

†P for interaction

Model 1: adjusted for age and sex

Model 2: Model 1 + LDL-C, baseline FPG, antihypertensive drugs, hypoglycemic drugs, hypertension and diabetes mellitus

Model 3: Model 2 + BMI, current smoking, current drinking, physical exercise and family history of cancer

Abbreviations: ARV, average real variability; BMI, body mass index; CI, confidence interval; FPG, fasting plasma glucose; HR, hazard ratio.
